# Soluble TIM-3, likely produced by myeloid cells, predicts resistance to immune checkpoint inhibitors in metastatic clear cell renal cell carcinoma

**DOI:** 10.1186/s13046-025-03293-y

**Published:** 2025-02-14

**Authors:** Ivan Pourmir, Nadine Benhamouda, Thi Tran, Hugo Roux, Joséphine Pineau, Alain Gey, Andyara Munoz, Nesrine Mabrouk, Nicolas Epaillard, Virginie Verkarre, Yann-Alexandre Vano, Eric Tartour, Stéphane Oudard

**Affiliations:** 1 Université Paris Cité, INSERM U970, PARCC, Paris, France; 2 Medical Oncology Department, Georges Pompidou European Hospital, CARPEM Cancer Institute, AP-HP Centre, Université Paris Cité, Paris, France; 3 Thoracic Oncology Department, Georges Pompidou European Hospital, CARPEM Cancer Institute, AP-HP Centre, Université Paris Cité, Paris, France; 4 Immunology Department, Georges Pompidou European Hospital, AP-HP Centre, Université Paris Cité, Paris, France; 5 Institut Curie, PSL University, Inserm U932, Immunity and Cancer, Paris, France; 6 Medical Oncology Department, Clinique Kuindo Magnin, Nouméa, Nouvelle Calédonie France; 7 Pathology Department, Georges Pompidou European Hospital, AP-HP Centre, Université Paris Cité, INSERM UMR970, Equipe Labellisée Ligue Contre Le Cancer, Paris, France; 8 Medical Oncology Department, Hôpital Foch, Suresnes, France; 9 Centre de Recherche Des Cordeliers, INSERM1138, Inflammation Complément Et Cancer, Université Paris Cité, Paris, France; 10 Paris – Cardiovascular Research Center, European Georges Pompidou Hospital, 56 Rue Leblanc, Paris, 75015 France

**Keywords:** Clear cell renal cell carcinoma, Soluble TIM-3, Myeloid cells, Biomarkers, Immunotherapy, Immune checkpoints, Nivolumab, Ipilimumab

## Abstract

**Background:**

Immunotherapies targeting PD-1 and CTLA-4 are key components of the treatment of metastatic clear cell renal cell carcinoma (mccRCC). However, they have distinct safety profiles and resistance to treatment can occur. We assess soluble TIM-3 (sTIM-3) in the plasma of mccRCC patients as a potential theranostic biomarker, as well as its source and biological significance.

**Methods:**

We analyzed the association between sTIM-3 and overall survival (OS), tumor response, and common clinical and biological factors in two mccRCC cohorts treated with anti-PD-1 (nivolumab, *n* = 27), anti-PD-1 or anti-PD-1 + anti-CTLA-4 (nivolumab + ipilimumab – N + I, *n* = 124). The origin and role of sTIM-3 are studied on tumor and blood samples, using multiplex immunohistochemistry and flow cytometry, as well as analyses of publicly available single-cell transcriptomic (scRNAseq) and mass cytometry data.

**Results:**

sTIM-3 is significantly elevated in the plasma of treatment-naive mccRCC. It shows distinct associations with survival on anti-PD-1 vs anti-PD-1 + anti-CTLA-4: under nivolumab monotherapy, sTIM-3-high patients have a significantly reduced survival compared to sTIM-3-low patients, while they have similar survival probabilities under N + I. sTIM-3 is independent from other clinical and biological factors. Myeloid immune cells appear as the prominent source of sTIM-3, which may indicate their dysfunctional role in the antitumor immune response.

**Conclusions:**

sTIM-3 appears to be a promising biomarker for optimizing treatment strategies in ccRCC as well as a potential therapeutic target, linked with to the immune myeloid compartment. Future investigations are warranted in patients treated with anti-PD-1 + antiangiogenic therapies.

**Supplementary Information:**

The online version contains supplementary material available at 10.1186/s13046-025-03293-y.

## Background

Immune checkpoint inhibitors (ICI) were introduced in clear cell renal cell carcinoma (ccRCC) as a second-line treatment in the metastatic setting (mccRCC) with the approval of nivolumab monotherapy, an antibody blocking the-programmed cell death protein-1 (PD-1) [1]. Anti-PD-1 were later combined in the first line with ipilimumab, an anti-cytotoxic T-lymphocyte-associated protein 4 (anti-CTLA-4) or antiangiogenic tyrosine kinase inhibitors (TKI) [2]. The International Metastatic RCC Database Consortium (IDMC) score is the only validated theranostic biomarker for mccRCC and previously helped selecting nivolumab + ipilimumab (N + I) over sunitinib [3, 4]. No predictive biomarkers exist to discern patients who may achieve maximal efficacy with N + I rather than with other anti-PD-1-based regimens.

T cell immunoglobulin and mucin domain-containing molecule 3 (TIM-3) is an immunomodulatory transmembrane protein classically ascribed as an exhaustion marker on T cells, conferring dysfunctionality [5]. In ccRCC, TIM-3-expressing T cells have been associated with poor prognosis [6, 7] and resistance to anti-PD-1 monotherapy [8, 9] or combined with antiangiogenics [10]. Clinical trials targeting TIM-3 in mccRCC are underway [11, 12]. Soluble isoforms of TIM-3 (sTIM-3) are generated through proteolytic cleavage and can be quantified in human plasma [13, 14]. Blood-based biomarkers have the advantages of ready accessibility and proximity to baseline measurements. We assessed whether plasma sTIM-3 is associated with resistance of mccRCC to ICI, given the detrimental value of TIM-3 expression in the TME [15] and sTIM-3 association with prognosis regardless of treatment [16].

Here we show that an acute sTIM-3 increase is induced via antitumor immunization in mice. We assess chronic plasma sTIM-3 elevation in mccRCC patients, its association with outcomes under ICI. We investigate the source of sTIM-3 by analyzing tumor specimens and peripheral blood mononuclear cells (PBMC) from mccRCC patients.

## Material and methods

### Patient cohorts and sample collection

ICI-treated mccRCC patients were participants from the Colcheckpoint (nivolumab monotherapy after prior antiangiogenic therapy) and BIONIKK (first line nivolumab monotherapy or N + I [17]) independent cohorts approved by the French Health authorities and ethics committee [CPP Ile-de-France 8 (ref.16.10.69) and CPP Ouest I SI CNRIPH n18.11.21.67518 respectively]. Healthy adult human samples were drawn from blood donations. Plasma and PBMC were collected immediately before ICI initiation for mccRCC (baseline). Multiplex immunohistochemistry (IHC) was performed on formalin-fixed, paraffin-embedded (FFPE) archival tissue of primary tumors from participants of the BIONIKK cohort. All the participants provided written informed consent.

### Mouse models

TC-1 cells were obtained from TC Wu's laboratory (John Hopkins Hospital) and injected into anesthetized animals. Mice were vaccinated with 200 µg G15F peptide from the HPV-16 E7 protein (Genosphere Biotechnologies) and 2 µg of α-galactosyl-ceramide (α-Gal-Cer) adjuvant (Funakoshi Co.).

CD8 + T cells depletion was performed in vivo by intraperitoneal injections of a depleting antibody (100 µg/100 µl anti-mouse CD8α rat IgG2bκ, clone 2.43 InVivoMab™, BioXCell).

Experiments were conducted on 8-week-old female C57BL/6 J mice (Janvier Labs) and were approved by the Ethics Committee of the University Paris Cité (CEEA34).

### sTIM-3 quantification

Human plasma samples were isolated by centrifugation and frozen at −80 °C for storage. After thawing the samples, sTIM-3 was quantified using the Luminex® Multiplex ProcartaPlex™ Human Immuno-Oncology Checkpoint Marker Panel 1 (14-Plex) kit (Thermo Fisher®), following the manufacturer’s instructions. Measurements were acquired using a BioPlex-200 (BioRad®).

sTIM-3 was quantified in mouse serum by ELISA (Mouse Tim-3 SimpleStep ELISA® kit—ab255721 Abcam®), absorbance was measured on a Spectrostar microplate reader and converted to concentrations using BMG Labtech software.

### Multiplex IHC

FFPE slides were incubated with specific antibodies (details in Supp. Table 2.A) and stained in a BOND-RX automate (Leica®) using Opal™ secondary antibodies and fluorescent reagents. The specificity of TIM-3 staining was confirmed in additional ccRCC samples using the corresponding isotype control. Images were acquired in a Vectra Polaris scanner (Akoya/PerkinElmer) at 20X magnification and analyzed using HALO™ (v.3.6.4, IndicaLab®).

### Flow cytometry

PBMC were isolated by Ficoll™ density gradient centrifugation and frozen for storage. PBMC were thawed, marked for viability (Zombie NIR, BioLegend®) and stained with antibodies against human markers (details in Supp. Table 2.B). Acquisition was performed on a Navios 10 colors cytometer (Beckman Coulter®), analyses were performed using Kaluza® (v 2.1). Thresholds for TIM-3 and CD14 positivity were set with the corresponding control isotypes.

### Single-cell RNA sequencing (scRNAseq) data analyses

ScRNAseq data from ccRCC and non-cancerous human kidneys were obtained from publicly available datasets [18–22] and analyzed in R (v.4.1.1) using Seurat (v.5.0.3). Cells were annotated through SingleR (v.1.8.1) and manually. Differential gene expression (DEG) and gene set enrichment analyses (GSEA) were performed using MAST (v.1.20.0) and enrichR (v.3.2). Pseudobulk analysis of scRNAseq data was performed using DESeq2 (v.1.34.0) on aggregated transcripts counts.

#### Statistical analysis

Comparisons were performed for quantitative continuous variables using Mann–Whitney U (a.k.a. Wilcoxon ranks sum), Kruskal–Wallis, paired Wilcoxon, Friedman and Student’s t tests. Appropriate tests were chosen depending on the distribution and the dependent/independent nature of the observations. Given the sufficient maturity of the data, Overall survival (OS) was chosen as the primary outcome for measuring ICI efficacy [23, 24]. OS probabilities were estimated using the Kaplan–Meier method, differences were quantified by Cox hazard ratios (HR) and tested using the log-rank test. Two-tailed *p*-values < 0.05 were considered statistically significant. *P*-value adjustments for multiple testing were performed using the Benjamini–Hochberg method for large-scale differential gene expression analyzes. In this case, adjusted *p*-values < 0.05 were considered to be statistically significant. sTIM3 values were batch-corrected through the ComBat() function from the sva R package (v. 3.50.0) prior to classification of individuals for comparisons between the nivolumab and N + I arms of the BIONIKK cohort (Supp. Figure 1). Analyses were performed using the R software (v.4.3.2; R Foundation for Statistical Computing, Vienna, Austria).

## Results

### sTIM-3 is elevated in mccRCC patients and associated with OS under ICI

**Fig. 1:**
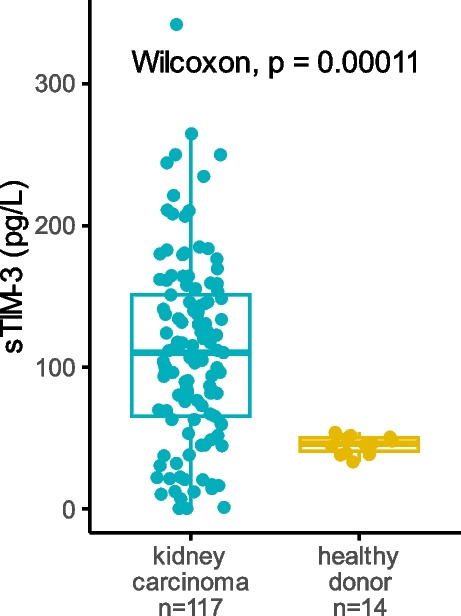
sTIM-3 plasma levels in ccRCC patients. sTIM-3 plasma levels were measured in treatment-naive ccRCC patients from the BIONIKK cohort before ICI treatment, and in healthy adults

Plasma sTIM-3 levels were significantly increased in treatment-naive mccRCC patients (median = 110.33 pg/L, IQR 65.36 to 151.10) from the BIONIKK cohort, compared to healthy adults (median = 46.10 pg/L, IQR 40.81 to 50.42, *p* < 10^–3^) (Fig. 1).

**Fig. 2:**
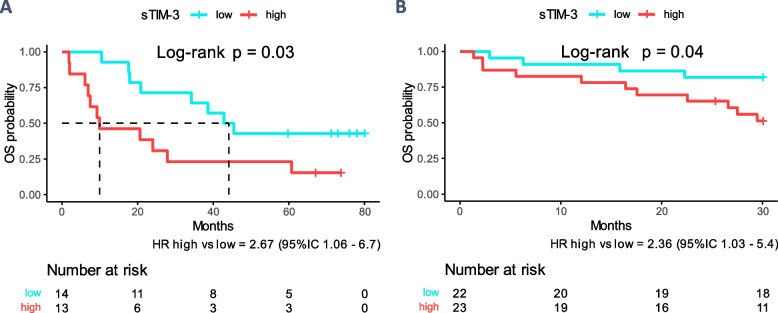
Association of sTIM-3 plasma levels with OS in ccRCC patients under anti-PD1. Patients were categorized in sTIM-3 high or low, depending on whether their individual values of plasma sTIM-3 fell below or above the median value within each cohort. OS was estimated using the Kaplan–Meier method and differences between sTIM-3-low and -high groups were tested with the log rank method. Patients’ characteristics are found in Supplementary Table 1. **A** Colcheckpoint cohort (*n* = 27). **B** BIONIKK cohort (*n* = 45)

High baseline sTIM-3 (above the median within each cohort) was associated with poor OS of ccRCC patients on nivolumab monotherapy, in both the Colcheckpoint (*n* = 27, HR for death sTIM-3-high vs sTIM-3-low = 2.67, *p* = 0.03) and BIONIKK (*n* = 45, HR sTIM-3-high vs sTIM-3-low = 2.36, *p* = 0.04) cohorts (Fig. 2A & B).

Interestingly, there was no OS difference according to sTIM-3 stratification in patients from the BIONIKK trial treated with N + I (*n* = 79, Supp. Figure 2.A). Furthermore, we found that sTIM-3-low participants in the nivolumab monotherapy and N + I arms had comparable OS, whereas for sTIM-3-high participants, N + I was significantly superior to nivolumab (Supp. Figure 2.B). To verify that the results in the N + I group were not confounded by IMDC scores (Supp. Table 1.C), a 2-variables Cox model for OS including IMDC and sTIM-3 categories was constructed. Still, the adjusted analysis showed no relationship between sTIM-3 category and OS under N + I (HR high vs low = 1.0, 95% IC 0.40—2.72) (Supp. Figure 3).

This suggests that sTIM-3 can distinguish mccRCC patients likely to achieve prolonged survival under anti-PD-1 monotherapy, whereas the anti-PD-1 + anti-CTLA4 combination retains its efficacy in sTIM-3-high patients.

### sTIM-3 is independent from clinical and biological prognostic markers

**Fig. 3:**
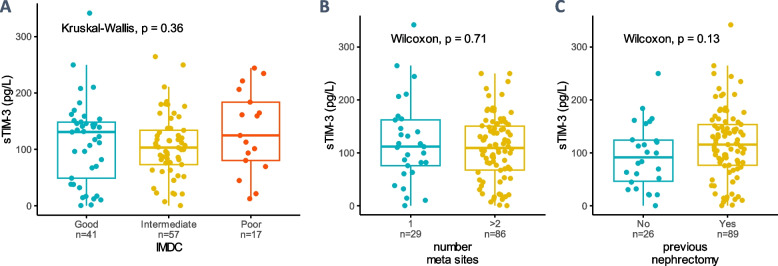
Comparison of sTIM-3 plasma levels in participants of BIONIKK (*n* = 133) categorized according to IMDC score and tumor burden. **A** sTIM-3 versus IMDC score calculated at baseline before ICI initiation. **B** sTIM-3 vs. the number of metastatic sites at baseline (1 site or ≥ 2 sites). **C** sTIM-3 vs. history of primary tumor removal (previous nephrectomy)

To assess the specificity of plasma sTIM-3 as a biomarker in the context of metastatic ccRCC, we tested its association with known baseline clinical and biological prognostic markers. IMDC categorization was not associated with sTIM-3 (Fig. 3A) and sTIM-3 was neither correlated with systemic inflammation markers included in the score (neutrophil and platelet counts, decreased hemoglobin) nor with other biomarkers (Supp. Figure 4 and Supp. Figure 6).

Unlike CRP (Supp. Figure 5), sTIM-3 was not associated with tumor burden proxies (number of metastatic sites or presence of primary tumor, Fig. 3B & C). Hence, sTim-3 does not simply reflect a higher tumor burden or systemic inflammation, it conveys additional information about the status of the disease. In addition, these results suggest a source other than tumor cells for sTIM-3.

### Plasma sTIM-3 is likely produced by myeloid cells in ccRCC

#### In situ multiplex IHC of ccRCC tumors

**Fig. 4:**
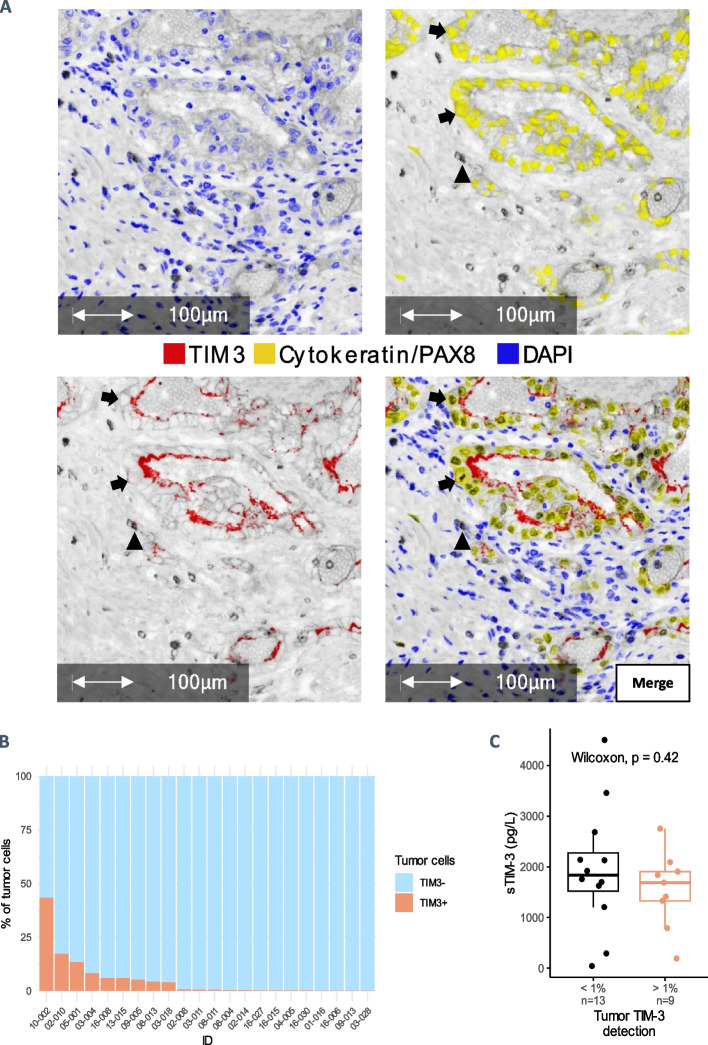
TIM-3 IHC detection on ccRCC tumors. 10 random tumor regions evenly spread on the slides were selected for analyzes. A custom phenotyping algorithm was used for quantification of total PAX8 + Cytokeratin + tumor cells and manual counting was performed for quantification of TIM3 + tumor cells. **A** Absorption view (× 20) of a ccRCC primary tumor FFPE sample analyzed with multiplex IHC. Blue: DAPI; Yellow: PanCytokeratine-PAX; Red: TIM-3. Arrows: TIM-3 positive tumor cells; Arrowhead: TIM-3 positive non-tumor cell. **B** % of TIM-3-positive tumor cells among tumor cells in each sample (*n* = 22). **C** Association of plasma sTIM-3 with TIM-3 tumor status

We assessed the expression of TIM-3 by tumor cells – defined as pancytokeratin and/or PAX8 positive cells—and tested its association with plasma sTIM-3 in a subset of BIONIKK participants (*n* = 22) (Fig. 4A).

There was high intra-tumor and inter-sample variability in TIM-3 staining of tumor cells (Fig. 4B), with a median of 0.34% (IQR 0.05% to 5.61%) positive tumor cells. Tumors with > 1% TIM-3 positive tumor cells were classified as TIM-3 positive. TIM-3 positivity was neither associated with higher levels of plasma sTIM-3 (Fig. 4C), nor with survival under nivolumab monotherapy (Supp. Figure 7).

The abundance of TIM-3-positive non-tumor cells did not appear to be correlated with plasma sTIM-3 (Spearman correlation = −0.26, *p* = 0.25, Supp. Figure 8). We further characterized non-tumor TIM-3 positive cells with additional broad immune markers (CD3, CD4, CD8 and CD68). Interestingly, a substantial proportion of intratumor myeloid cells was TIM-3 positive. They seemed to be overall at least as abundant as TIM-3 positive lymphoid cells (Supp. Figure 9). Nonetheless, no direct correlation between the abundance of the different TIM-3 positive immune populations and sTIM-3 plasma levels was evidenced (not shown). This might be partly due to the sensitivity of IHC to the shedding of TIM-3 (see Discussion).

#### scRNAseq of ccRCC tumors

**Fig. 5:**
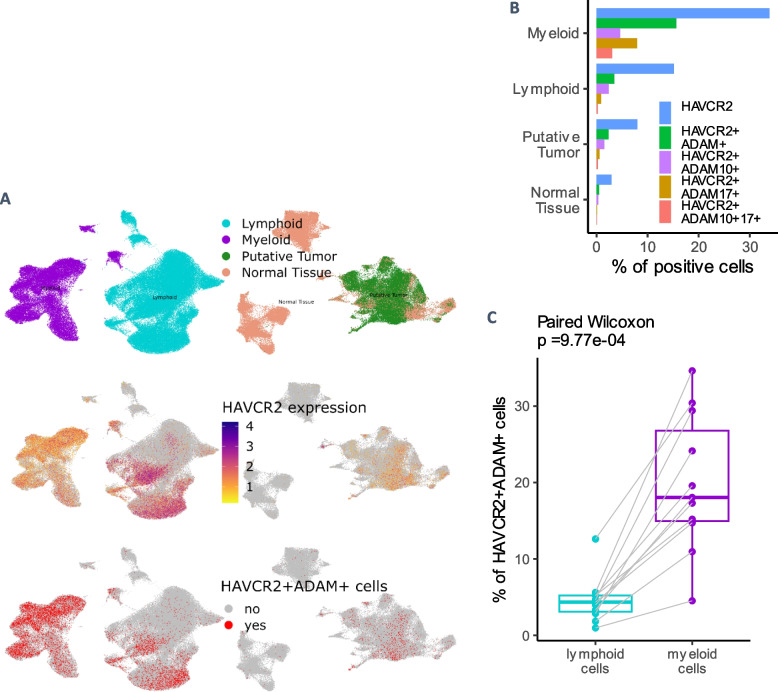
Obradovic et al. ccRCC scRNAseq dataset. **A** Tumor and adjacent tissue samples. Upper section: UMAP of cell lineages; middle section: HAVCR2 expression; lower section: HAVCR2 + ADAM + double-positive cells repartition. **B** % of cells in the pooled dataset expressing HAVCR2 and ADAM10 and/or ADAM17 in broad lineages. HAVCR2 + ADAM10 +—detection of ADAM10 transcripts but not ADAM17; HAVCR2 + ADAM17 +—detection of ADAM17 transcripts but not ADAM10, HAVCR2 + ADAM10 + 17 +—detection of both metalloproteinases. **C** Comparison of the proportions of HAVCR2 + ADAM + cells within the lymphoid and myeloid lineages, for each patient in tumor samples

The main hypothesis for the production of sTIM-3 in human is its shedding from double-positive HAVCR2 + ADAM + cells, co-expressing HAVCR2 (encoding TIM-3) and one or both of the metalloproteinases (ADAM10 and ADAM17) for which the cleaving activity of membrane TIM-3 is reported [13, 14]. We quantified the expression of these genes in several scRNAseq datasets of ccRCC and healthy kidney samples, the results are shown in Obradovic et al. dataset (Fig. 5).

Although some CD8 + and NK lymphocyte clusters had high transcription levels of HAVCR2, myeloid clusters (Supp. Fig. 10,11,12,13) were identified as the most enriched lineage in HAVCR2 + ADAM + cells, with even distributions of the three different double-positive types (Fig. 5B; Supp. Fig. 10 & 12). HAVCR2 expression was detected in renal tissue clusters (tumor and healthy samples) but they contained marginal proportions of HAVCR2 + ADAM + cells (Fig. 5A & B, Supp. Fig. 11 & 12). Consistent findings were reproduced in the three other scRNAseq datasets.

HAVCR2 + ADAM + enriched monocytes clusters were common to paired tumors and PBMC in a dataset of 3 ccRCC patients [20]. Consistent with our flow cytometry data, HAVCR2 expression in PBMC was mainly found in monocytes and NK clusters but was quasi-undetectable in T cells (Supp. Fig. 13).

Principal component analysis (PCA) of the top 200 most variable genes showed a clear separation of double-positive and non-double-positive pseudobulk samples generated from Obradovic et al. dataset, despite technical batch variations, suggesting a distinct transcriptomic state for HAVCR2 + ADAM + myeloid cells (Supp. Fig. 14).

A differential gene expression profile comparing HAVCR2 + ADAM + myeloid cells with other myeloid cells was established using Seurat implementation of the MAST algorithm. Among the top DEG, the putative pro-tumor markers TREM2 and APOE were found increased in HAVCR2 + ADAM + myeloid cells (Khantakova, Brioschi, et Molgora 2022; Bancaro et al. 2023), while NLRP3 was decreased, which may be linked to decreased APC functionality through inflammasomes (Dixon et al. 2021). GSEA suggested global downregulation of proteins synthesis in HAVCR2 + ADAM + myeloid cells (Supp. Fig. 15), as wells as cellular respiration (oxidative phosphorylation).

These results favor the hypothesis of myeloid cells being the major source of sTIM3 in mccRCC. sTIM-3 could reflect the presence of dysfunctional HAVCR2 + ADAM + myeloid cells with globally decreased anabolic activity [25].

#### Mass cytometry of ccRCC tumors

In order to quantify the expression of TIM-3 by the different cell lineages of the ccRCC TME at the protein level, we reanalyzed publicly available mass cytometry data of 72 ccRCC tumors and 5 healthy kidney samples published by Chevrier et al. [26]. TIM-3 appeared to be fairly expressed by lymphoid and myeloid cells but much less expressed by non-immune cells (Supp. Fig. 16 & 17). There was a significant difference in median signal intensity depending on the cell lineage (*p* < 0.0001), TIM-3 detection was generally higher for lymphoid and myeloid cells than for non-immune cells in all ccRCC samples (*p* = 5.33 × 10^–13^ and 1.15 × 10^–12^ respectively), in line with our findings on the scRNAseq datasets (Supp. Figure 16.B). Notably, the median TIM-3 signal intensity was null in non-immune cells of kidney samples from healthy donors (Supp. Figure 16.C).

#### PBMC cytometry of ccRCC patients

**Fig. 6:**
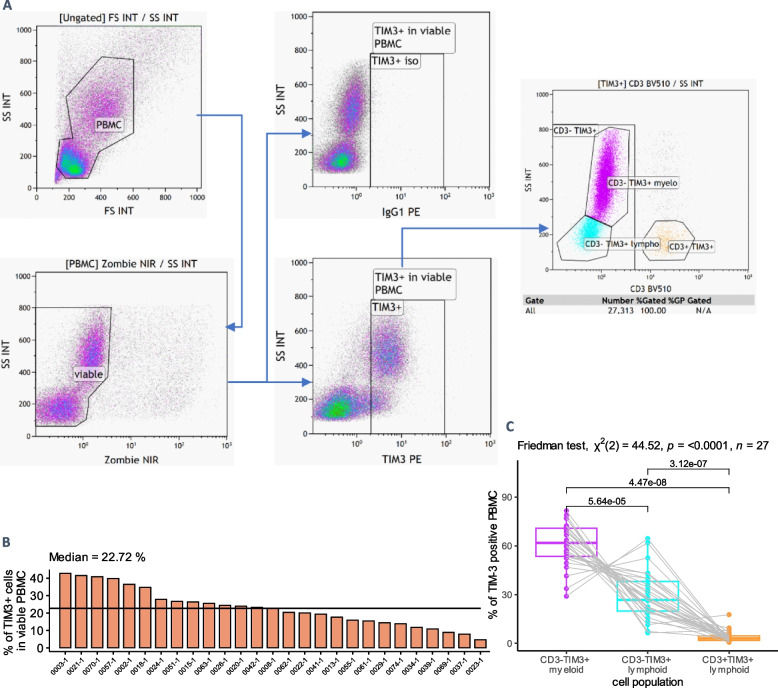
Flow cytometry quantification of TIM-3-positive (TIM3 +) cells in PBMC of ccRCC patients (Colcheckpoint cohort, *n* = 27). **A** Gating strategy; we considered CD3-SSC-A^high^ cells as myeloid cells, CD3 + SSC-A^low^ as T cells and CD3-SSC-A^low^ as non-T lymphoid cells. **B** Percentage of TIM3 + cells within PBMC of Colcheckpoint participants. **C **Proportion of CD3- myeloid, CD3- lymphoid and CD3 + lymphoid cells within TIM3 + cells in PBMC of Colcheckpoint participants

We quantified TIM-3-positive (TIM3 +) cells in PBMC from mccRCC patients (Fig. 6A), their median proportion was 22.7% (IQR 14.9% to 27.3%) (Fig. 6B).

CD3-TIM3 + myeloid cells were the most common type of TIM3 + PBMC (median 61.9% of TIM3 + PBMC), followed by CD3-TIM3 + lymphoid cells. Surprisingly, T cells (CD3 + TIM3 +) accounted for a minority of TIM3 + PBMC (Fig. 6C). Given their intermediate CD4 positivity (Supp. Figure 20) and that their proportion among TIM3 + PBMC was the same as that of CD14 + TIM3 + PBMC on a separate panel (Supp. Figure 21), we hypothesized that most CD3-TIM3 + myeloid cells had a monocytic origin. CD3-TIM3 + lymphoid PBMC were most likely NK cells, given their CD3-CD8low phenotype and that B cells (quantified with CD19 on another panel, not shown) were not found in TIM3 + PBMC. These results are consistent with data from the literature on peripheral blood cells from healthy donors, showing TIM-3 detection on a high proportion of monocytes, a moderate proportion of NK cells, and low or absent detection on T cells and granulocytes (Kikushige et al. 2010; [13]).

## Discussion

Plasma sTIM-3 is a promising blood-based biomarker associated with ICI efficacy in mccRCC, with differential effects on nivolumab versus N + I, and is independent of common clinical or inflammatory markers. An association with the efficacy of current anti-PD-1 + TKI regimens is also plausible and will be investigated in future research. If a differential effect on N + I compared to anti-PD-1 + TKI is confirmed, this could make sTIM-3 a theranostic biomarker. Defining an sTIM-3-high population for which the addition of anti-CTLA-4 is needed to overcome resistance to anti-PD-1 would facilitate the indication of N + I, for which the benefit-risk balance is delicate. sTIM-3 is also promising for other cancers where anti-PD-1 monotherapies are challenged by combinations, without strong decisional criteria (e.g. melanoma and lung cancer). Of note, slightly higher plasma sTIM-3 levels were found in men (median = 117.21 pg/L in men vs. 87.59 pg/L in women, unadjusted *p* = 0.048, Supp. Figure 6.B). If a true association between sex and sTIM-3 levels exists, to our knowledge, there is no obvious mechanism to explain this result and it is unlikely to affect the predictive value of sTIM-3.

TIM-3 is known for its inhibitory role at the membrane of T cells, its shedding into sTIM-3 by activated CD8 + T cells has been reported [14]. Unexpectedly, our study suggests that myeloid cells are the major source of sTIM-3 in mccRCC. TIM-3 was found on tumor-associated macrophages (TAM) and associated with PFS, in another cohort of ccRCC, and was induced on monocytes co-cultured with RCC cell-lines. However, the patients were not treated with ICI and monocytes were obtained from healthy volunteers [27]. LPS-activated monocytes from healthy donors and myeloid leukemia cells shed sTIM-3 upon activation of ADAMs [13, 28]. In mccRCC, we show that myeloid cells represent the majority of TIM-3-positive PBMC and the most enriched lineage in HAVCR2-expressing cells within the TME. It must be noted that the 2E2 mAb used by Chevrier et al. and by us to detect TIM-3 in cytometry experiments most likely binds to a part of TIM-3’s ectodomain that is shed [13, 14]. Nevertheless, our analyses of scRNAseq data showed more co-expression of ADAM10/17 in myeloid cells from the TME and PBMC samples compared to lymphoid cells. A bias leading to underestimation of TIM-3 expression from its membrane detection, because of the shedding of the marker, is hence more likely to occur for myeloid cells, especially in the TME where the activity of ADAM is known to increase [29]. Consistent with this hypothesis, the median signal intensity for TIM-3 was higher in lymphoid cells than in myeloid cells in most ccRCC samples from Chevrier et al. dataset (Supp. Figure 16.B, Supp. Fig. 17). Besides, TIM-3 was detected at the apical border of some healthy and cancerous proximal tubule cells in IHC experiments (Fig. 4A). One hypothesis is that sTIM-3 undergoes glomerular filtration with re-uptake of the antigen by these cells [30]. Still, TIM-3 appeared to be fairly expressed by immune cells but with null median signal intensities for non-immune cells in most samples of Chevrier et al. dataset (Supp. Fig. 16.B & C). Finally, granulocytes are quasi-absent from PBMC and from the scRNAseq datasets we reanalyzed but their role as source of sTIM-3 cannot be excluded. Nevertheless, it can be noted that existing studies from the literature report no TIM-3 expression on peripheral granulocytes from healthy adult donors, unlike monocytes (Kikushige et al. 2010b; [13],Hakim et al. 2020; H. Wang et al. 2022).

A secreted sTIM-3 isoform is derived from the alternative splicing of murine TIM-3 mRNA [31]. Nonetheless, mouse embryonic fibroblasts shed transfected human TIM-3 and murine TIM-2 (TIM family) via ADAM10/17, supporting the relevance of studying sTIM-3 in this species [13, 32]. To assess sTIM-3 at the early stage of the tumor response, we used a syngeneic TC-1 mouse tumor model, which is spontaneously poorly immunogenic but for which a specific antitumor immune response can be triggered via vaccination. Control mice solely inoculated with TC-1 cells showed no significant variation in plasma sTIM-3 18 days after the graft, whereas vaccinated mice exhibited a significant increase (Supp. Figure 22). This suggests a prominent role of the immune response in the production of sTIM-3 in this model, rather than the growth of the tumor burden per se. Moreover, in a second experiment, sTIM-3 increase upon antitumor immunization was found at earlier time-points, as soon as 4 days after the vaccine administration (e.g. 11 days after tumor graft) and was not modified by CD8 + T cells depletion (Supp. Figure 23). The timing of sTIM-3 increase and its sustainability despite CD8 + T cells elimination favor the hypothesis of the early release of sTIM-3 by innate immune cells, possibly myeloid cells, in this model. Confirming experiments of myeloid cells depletion are being conducted in this model.

Regarding the biological meaning of sTIM-3 in mccRCC patients, there are two (non-exclusive) possibilities: On one hand, sTIM-3 could have per se a pro-tumor and/or immunosuppressive effect. For instance, it has been shown that sTIM-3 inhibits interleukin-2 expression, which is known for its major role in T cell immunity and as one of the first immunotherapies with antitumor activity in mccRCC [28, 33]. On the other hand, plasma sTIM-3 could be a marker of pro-tumor processes or cell types, such as ADAM10/17 activity in the TME [34, 35]. McDermott et al. reported an association between high expression of a gene set related to myeloid inflammation and reduced PFS of metastatic ccRCC patients treated with atezolizumab (anti-PD-L1), alone or combined with bevacizumab, in the IMmotion150 trial [36]. Recently, Vanmeerbeek et al. identified TAM co-expressing TIM-3 and VSIR, associated with resistance to ICI. Interestingly, HAVCR2 was one of the most transcriptionally enriched genes in myeloid cells from ICI-unresponsive patients in their cross-cancer dataset [37]. We show that HAVCR2 + ADAM + myeloid cells have increased transcription of TREM2 and APOE, which have been proposed as pro-tumor macrophage markers [38]. The release of sTIM-3 may also reflect a deleterious state of over-activation of myeloid APC, which are required to prime T cells and sustain the response to ICI [39–41]. In an experimental mouse model of systemic inflammatory response syndrome, intravenous injection of LPS, which is also known to induce sTIM-3 release by monocytes, could recreate a state of immune paralysis [42]. GSEA suggest that HAVCR2 + ADAM + myeloid APC have globally impaired transcriptional and translational capacities (Supp. Figure 15) [25, 43].

Understanding the biological meaning of plasma sTIM-3 will be crucial for TIM-3-targeted therapies: According to the relevant hypotheses, strategies to treat sTIM-3 high patients would be to either block the putative deleterious action of sTIM-3, inhibit ADAMs’ activities, deplete TIM-3 + pro-tumor myeloid cells or restore the function of paralyzed APC.

## Conclusion

- Plasma sTIM-3 is elevated in mccRCC and independent of clinical and inflammatory prognostic markers. It is a promising blood-based biomarker associated with ICI efficacy in this setting.
- The immune myeloid compartment is a predominant candidate as the source of plasma sTIM-3 in mccRCC.
- Further functional studies will precise the biological role of sTIM-3 in mccRCC and the ensuing therapeutic targets.

## Supplementary Information

Supplementary Material 1.

## Data Availability

The human datasets generated during the current study are not publicly available due to the local regulation on patients’ data protection. Requests for anonymized patient data will be examined on an individual basis by relevant administration committees of the Colcheckpoint and BIONIKK cohorts’ data. The processed data generated by the authors from animal experiments are available upon request from the corresponding authors. The R code generated by the authors for bioinformatics and statistical analyses is available upon request from the corresponding authors. The publicly archived scRNAseq datasets reanalyzed during the current study are available at the following addresses: https://github.com/Aleksobrad/single-cell-rcc-pipeline; https://singlecell.broadinstitute.org/single_cell/study/SCP1288/tumor-and-immune-reprogramming-during-immunotherapy-in-advanced-renal-cell-carcinoma#study-summary; https://github.com/ncborcherding/ccRCC; and http://www.kidneycellatlas.org/. The publicly archived mass cytometry dataset reanalyzed during the current study is available at the following address: https://premium.cytobank.org/cytobank/projects/875.

## Abbreviations

ADAM10: A disintegrin and metalloproteinase domain 10
ADAM17: A disintegrin and metalloproteinase domain 17
APC: Antigen presenting cells
APOE: Apolipoprotein E
ccRCC: Clear cell renal cell carcinoma
CD: Cluster of differentiation
CRP: C-reactive protein
CTLA-4: Cytotoxic t-lymphocyte-associated protein 4
FFPE: Formalin-fixed, paraffin-embedded
GAL-9: Galectin-9
GSEA: Gene set enrichment analysis
HAVCR2: Hepatitis a virus cellular receptor 2
HPV-16: Human papillomavirus 16
HR: Hazard ratio
IC: Immune checkpoints
ICI: Immune checkpoint inhibitors
IFN: Interferon
IHC: Immunohistochemistry
IL: Interleukin
IMDC: International metastatic rcc database consortium
IQR: Interquartile ranges
LPS: Lipopolysaccharide
mAb: Monoclonal antibodies
MFI: Median fluorescence intensity
MHC: Major histocompatibility complex
NK: Natural killer
NLR: Neutrophil-to-lymphocyte ratio
NLRP3: Nlr family pyrin domain containing 3
NSCLC: Non-small cell lung cancer
ORR: Overall response rate
OS: Overall survival
PAX8: Paired box 8
PBMC: Peripheral blood mononuclear cells
PD-1: Programmed cell death protein 1
PFS: Progression-free survival
PS: Performance status
sADAM10: Soluble ADAM10
sADAM17: Soluble ADAM17
scRNAseq: Single-cell RNA sequencing
TAM: Tumor associated macrophages
TCGA: The cancer genome genome atlas
TCR: T cell receptor
TILs: Tumor-infiltrating lymphocytes
TIM-3: T cell immunoglobulin and mucin domain-containing molecule 3
TKI: Tyrosine kinase inhibitors
TME: Tumor microenvironment
Tregs: Regulatory T cells
TREM2: Triggering receptor expressed on myeloid cells 2

## References

[1] FDA C for DE and. Approved Drugs - Nivolumab (Opdivo Injection) - advanced renal cell carcinoma. Center for Drug Evaluation and Research. 2015. Available from: http://wayback.archive-it.org/7993/20170111231614/http://www.fda.gov/Drugs/InformationOnDrugs/ApprovedDrugs/ucm474092.htm. Cited 18 Sep 2023.

[2] Powles T, Albiges L, Bex A, Comperat E, Grünwald V, Kanesvaran R, Kitamura H, McKay R, Porta C, Procopio G, Schmidinger M, Suarez C, Teoh J, de Velasco G, Young M, Gillessen S; ESMO Guidelines Committee. Electronic address: clinicalguidelines@esmo.org. Renal cell carcinoma: ESMO Clinical Practice Guideline for diagnosis, treatment and follow-up. Ann Oncol. 2024;35(8):692–706. 10.1016/j.annonc.2024.05.537. Epub 2024 May 22 10.1016/j.annonc.2024.05.537 38788900

[3] Heng DY, Xie W, Regan MM, Harshman LC, Bjarnason GA, Vaishampayan UN, et al. External validation and comparison with other models of the international metastatic renal-cell carcinoma database consortium prognostic model: a population-based study. Lancet Oncol. 2013;14(2):141–8. 23312463 10.1016/S1470-2045(12)70559-4 PMC4144042

[4] Motzer RJ, Rini BI, McDermott DF, Arén Frontera O, Hammers HJ, Carducci MA, et al. Nivolumab plus ipilimumab versus sunitinib in first-line treatment for advanced renal cell carcinoma: extended follow-up of efficacy and safety results from a randomised, controlled, phase 3 trial. Lancet Oncol. 2019;20(10):1370–85. 31427204 10.1016/S1470-2045(19)30413-9 PMC7497870

[5] Tang R, Rangachari M, Kuchroo VK. Tim-3: A co-receptor with diverse roles in T cell exhaustion and tolerance. Semin Immunol. 2019;42:101302. 31604535 10.1016/j.smim.2019.101302

[6] Granier C, Dariane C, Combe P, Verkarre V, Urien S, Badoual C, et al. Tim-3 expression on tumor-infiltrating PD-1+CD8+ T cells correlates with poor clinical outcome in renal cell carcinoma. Cancer Res. 2017;77(5):1075–82. 27872087 10.1158/0008-5472.CAN-16-0274

[7] Hu J, Chen Z, Bao L, Zhou L, Hou Y, Liu L, et al. Single-cell transcriptome analysis reveals intratumoral heterogeneity in ccRCC, which results in different clinical outcomes. Mol Ther. 2020;28(7):1658–72. 32396851 10.1016/j.ymthe.2020.04.023 PMC7335756

[8] Pignon JC, Jegede O, Shukla SA, Braun DA, Horak CE, Wind-Rotolo M, et al. irRECIST for the evaluation of candidate biomarkers of response to nivolumab in metastatic clear cell renal cell carcinoma: analysis of a phase II prospective clinical trial. Clin Cancer Res Off J Am Assoc Cancer Res. 2019;25(7):2174–84. 10.1158/1078-0432.CCR-18-3206 PMC6445699 30670497

[9] Ficial M, Jegede OA, Sant’Angelo M, Hou Y, Flaifel A, Pignon JC, et al. Expression of T-Cell exhaustion molecules and human endogenous retroviruses as predictive biomarkers for response to nivolumab in metastatic clear cell renal cell carcinoma. Clin Cancer Res. 2021;27(5):1371–80. 33219016 10.1158/1078-0432.CCR-20-3084 PMC8443005

[10] Saliby RM, El Zarif T, Bakouny Z, Shah V, Xie W, Flippot R, et al. Circulating and intratumoral immune determinants of response to atezolizumab plus bevacizumab in patients with variant histology or sarcomatoid renal cell carcinoma. Cancer Immunol Res. 2023;11(8):1114–24. 37279009 10.1158/2326-6066.CIR-22-0996 PMC10526700

[11] Tian T, Li Z. Targeting Tim-3 in Cancer With Resistance to PD-1/PD-L1 Blockade. Front Oncol. 2021;11:731175. 34631560 10.3389/fonc.2021.731175 PMC8492972

[12] Cai L, Li Y, Tan J, Xu L, Li Y. Targeting LAG-3, TIM-3, and TIGIT for cancer immunotherapy. J Hematol OncolJ Hematol Oncol. 2023;16(1):101. 37670328 10.1186/s13045-023-01499-1 PMC10478462

[13] Möller-Hackbarth K, Dewitz C, Schweigert O, Trad A, Garbers C, Rose-John S, et al. A disintegrin and metalloprotease (ADAM) 10 and ADAM17 are major sheddases of T cell immunoglobulin and mucin domain 3 (Tim-3). J Biol Chem. 2013;288(48):34529–44. 24121505 10.1074/jbc.M113.488478 PMC3843067

[14] Clayton KL, Douglas-Vail MB, Nur-ur Rahman AKM, Medcalf KE, Xie IY, Chew GM, et al. Soluble T cell immunoglobulin mucin domain 3 is shed from CD8+ T cells by the sheddase ADAM10, is increased in plasma during untreated HIV infection, and correlates with HIV disease progression. J Virol. 2015;89(7):3723–36. 25609823 10.1128/JVI.00006-15 PMC4403393

[15] Giraldo NA, Becht E, Vano Y, Petitprez F, Lacroix L, Validire P, et al. Tumor-infiltrating and peripheral blood T-cell immunophenotypes predict early relapse in localized clear cell renal cell carcinoma. Clin Cancer Res Off J Am Assoc Cancer Res. 2017;23(15):4416–28. 10.1158/1078-0432.CCR-16-2848 28213366

[16] Wang Q, Zhang J, Tu H, Liang D, Chang David W, Ye Y, et al. Soluble immune checkpoint-related proteins as predictors of tumor recurrence, survival, and T cell phenotypes in clear cell renal cell carcinoma patients. J Immunother Cancer. 2019;7(1):334. 31783776 10.1186/s40425-019-0810-y PMC6884764

[17] Vano YA, Elaidi R, Bennamoun M, Chevreau C, Borchiellini D, Pannier D, et al. Nivolumab, nivolumab–ipilimumab, and VEGFR-tyrosine kinase inhibitors as first-line treatment for metastatic clear-cell renal cell carcinoma (BIONIKK): a biomarker-driven, open-label, non-comparative, randomised, phase 2 trial. Lancet Oncol. 2022;23(5):612–24. 35390339 10.1016/S1470-2045(22)00128-0

[18] Obradovic A, Chowdhury N, Haake SM, Ager C, Wang V, Vlahos L, et al. Single-cell protein activity analysis identifies recurrence-associated renal tumor macrophages. Cell. 2021;184(11):2988-3005.e16. 34019793 10.1016/j.cell.2021.04.038 PMC8479759

[19] Bi K, He MX, Bakouny Z, Kanodia A, Napolitano S, Wu J, et al. Tumor and immune reprogramming during immunotherapy in advanced renal cell carcinoma. Cancer Cell. 2021;39(5):649-661.e5. 33711272 10.1016/j.ccell.2021.02.015 PMC8115394

[20] Borcherding N, Vishwakarma A, Voigt AP, Bellizzi A, Kaplan J, Nepple K, et al. Mapping the immune environment in clear cell renal carcinoma by single-cell genomics. Commun Biol. 2021;4. Available from: https://www.ncbi.nlm.nih.gov/pmc/articles/PMC7840906/. Cited 15 Feb 2021. 10.1038/s42003-020-01625-6 PMC7840906 33504936

[21] Braun DA, Street K, Burke KP, Cookmeyer DL, Denize T, Pedersen CB, et al. Progressive immune dysfunction with advancing disease stage in renal cell carcinoma. Cancer Cell. 2021;39(5):632-648.e8. 33711273 10.1016/j.ccell.2021.02.013 PMC8138872

[22] Stewart BJ, Ferdinand JR, Young MD, Mitchell TJ, Loudon KW, Riding AM, et al. Spatio-temporal immune zonation of the human kidney. Science. 2019;365(6460):1461–6. 31604275 10.1126/science.aat5031 PMC7343525

[23] Borcoman E, Kanjanapan Y, Champiat S, Kato S, Servois V, Kurzrock R, et al. Novel patterns of response under immunotherapy. Ann Oncol. 2019;30(3):385–96. 30657859 10.1093/annonc/mdz003

[24] Kluger HM, Tawbi HA, Ascierto ML, Bowden M, Callahan MK, Cha E, et al. Defining tumor resistance to PD-1 pathway blockade: recommendations from the first meeting of the SITC immunotherapy resistance taskforce. J Immunother Cancer. 2020;8(1):e000398. 32238470 10.1136/jitc-2019-000398 PMC7174063

[25] Liu X, Han W, Hu X. Chapter Three - Post-transcriptional regulation of myeloid cell-mediated inflammatory responses. In: Alt FW, Murphy KM, editors. Advances in Immunology. Academic Press; 2023. p. 59–82. (Advances in Immunology; vol. 160). Available from: https://www.sciencedirect.com/science/article/pii/S0065277623000299. Cited 18 Dec 2023 . 10.1016/bs.ai.2023.09.001 38042586

[26] Chevrier S, Levine JH, Zanotelli VRT, Silina K, Schulz D, Bacac M, et al. An immune atlas of clear cell renal cell carcinoma. Cell. 2017;169(4):736-749.e18. 28475899 10.1016/j.cell.2017.04.016 PMC5422211

[27] Komohara Y, Morita T, Annan DA, Horlad H, Ohnishi K, Yamada S, et al. The coordinated actions of TIM-3 on cancer and myeloid cells in the regulation of tumorigenicity and clinical prognosis in clear cell renal cell carcinomas. Cancer Immunol Res. 2015;3(9):999–1007. 25783986 10.1158/2326-6066.CIR-14-0156

[28] Gonçalves Silva I, Yasinska IM, Sakhnevych SS, Fiedler W, Wellbrock J, Bardelli M, et al. The Tim-3-galectin-9 secretory pathway is involved in the immune escape of human acute myeloid leukemia cells. eBioMedicine. 2017;22:44–57. 28750861 10.1016/j.ebiom.2017.07.018 PMC5552242

[29] Herrlich P, Herrlich A. ADAM metalloprotease-released cancer biomarkers. Trends Cancer. 2017;3(7):482–90. 28718403 10.1016/j.trecan.2017.05.001

[30] Nielsen R, Christensen EI, Birn H. Megalin and cubilin in proximal tubule protein reabsorption: from experimental models to human disease. Kidney Int. 2016;89(1):58–67. 26759048 10.1016/j.kint.2015.11.007

[31] Sabatos CA, Chakravarti S, Cha E, Schubart A, Sánchez-Fueyo A, Zheng XX, et al. Interaction of Tim-3 and Tim-3 ligand regulates T helper type 1 responses and induction of peripheral tolerance. Nat Immunol. 2003;4(11):1102–10. 14556006 10.1038/ni988

[32] Dewitz C, Möller-Hackbarth K, Schweigert O, Reiss K, Chalaris A, Scheller J, et al. T-cell immunoglobulin and mucin domain 2 (TIM-2) is a target of ADAM10-mediated ectodomain shedding. FEBS J. 2014;281(1):157–74. 24164679 10.1111/febs.12583

[33] Fyfe G, Fisher RI, Rosenberg SA, Sznol M, Parkinson DR, Louie AC. Results of treatment of 255 patients with metastatic renal cell carcinoma who received high-dose recombinant interleukin-2 therapy. J Clin Oncol. 1995;13(3):688–96. 7884429 10.1200/JCO.1995.13.3.688

[34] Black RA, Rauch CT, Kozlosky CJ, Peschon JJ, Slack JL, Wolfson MF, et al. A metalloproteinase disintegrin that releases tumour-necrosis factor-α from cells. Nature. 1997;385(6618):729–33. 9034190 10.1038/385729a0

[35] Ivetic A, Hoskins Green HL, Hart SJ. L-selectin: a major regulator of leukocyte adhesion. Migration Signaling Front Immunol. 2019;10:1068. 31139190 10.3389/fimmu.2019.01068 PMC6527602

[36] McDermott DF, Huseni MA, Atkins MB, Motzer RJ, Rini BI, Escudier B, et al. Clinical activity and molecular correlates of response to atezolizumab alone or in combination with bevacizumab versus sunitinib in renal cell carcinoma. Nat Med. 2018;24(6):749–57. 29867230 10.1038/s41591-018-0053-3 PMC6721896

[37] Vanmeerbeek I, Naulaerts S, Sprooten J, Laureano RS, Govaerts J, Trotta R, et al. Targeting conserved TIM3+VISTA+ tumor-associated macrophages overcomes resistance to cancer immunotherapy. Sci Adv. 2024;10(29):eadm8660. 39028818 10.1126/sciadv.adm8660 PMC11259173

[38] Khantakova D, Brioschi S, Molgora M. Exploring the impact of TREM2 in tumor-associated macrophages. Vaccines. 2022;10(6):943. 35746551 10.3390/vaccines10060943 PMC9227554

[39] Magen A, Hamon P, Fiaschi N, Soong BY, Park MD, Mattiuz R, et al. Intratumoral dendritic cell–CD4+ T helper cell niches enable CD8+ T cell differentiation following PD-1 blockade in hepatocellular carcinoma. Nat Med. 2023;29(6):1389–99. 37322116 10.1038/s41591-023-02345-0 PMC11027932

[40] Chen JH, Nieman LT, Spurrell M, Jorgji V, Elmelech L, Richieri P, et al. Human lung cancer harbors spatially organized stem-immunity hubs associated with response to immunotherapy. Nat Immunol. 2024;25(4):644–58. 38503922 10.1038/s41590-024-01792-2 PMC12096941

[41] Espinosa-Carrasco G, Chiu E, Scrivo A, Zumbo P, Dave A, Betel D, et al. Intratumoral immune triads are required for immunotherapy-mediated elimination of solid tumors. Cancer Cell. 2024 . Available from: https://www.sciencedirect.com/science/article/pii/S1535610824001934. Cited 2 Jul 2024. 10.1016/j.ccell.2024.05.025 PMC11413804 38906155

[42] Ashayeripanah M, Vega-Ramos J, Fernandez-Ruiz D, Valikhani S, Lun ATL, White JT, et al. Systemic inflammatory response syndrome triggered by blood-borne pathogens induces prolonged dendritic cell paralysis and immunosuppression. Cell Rep. 2024;43(2). Available from: https://www.cell.com/cell-reports/abstract/S2211-1247(24)00082-2. Cited 13 Aug 2024. 10.1016/j.celrep.2024.113754 38354086

[43] Roquilly A, Jacqueline C, Davieau M, Mollé A, Sadek A, Fourgeux C, et al. Alveolar macrophages are epigenetically altered after inflammation, leading to long-term lung immunoparalysis. Nat Immunol. 2020;21(6):636–48. 32424365 10.1038/s41590-020-0673-x

