# Supplementary material for: Soluble TIM-3, likely produced by myeloid cells, predicts resistance to immune checkpoint inhibitors in metastatic clear cell renal cell carcinoma
Source: J Exp Clin Cancer Res. 2025 Feb 14;44:54. doi: 10.1186/s13046-025-03293-y (PMC11827183; doi:10.1186/s13046-025-03293-y)
Supplement: Supplementary file 1 — Supplementary Material 1. [file 13046_2025_3293_MOESM1_ESM.pdf]

Supplementary figures

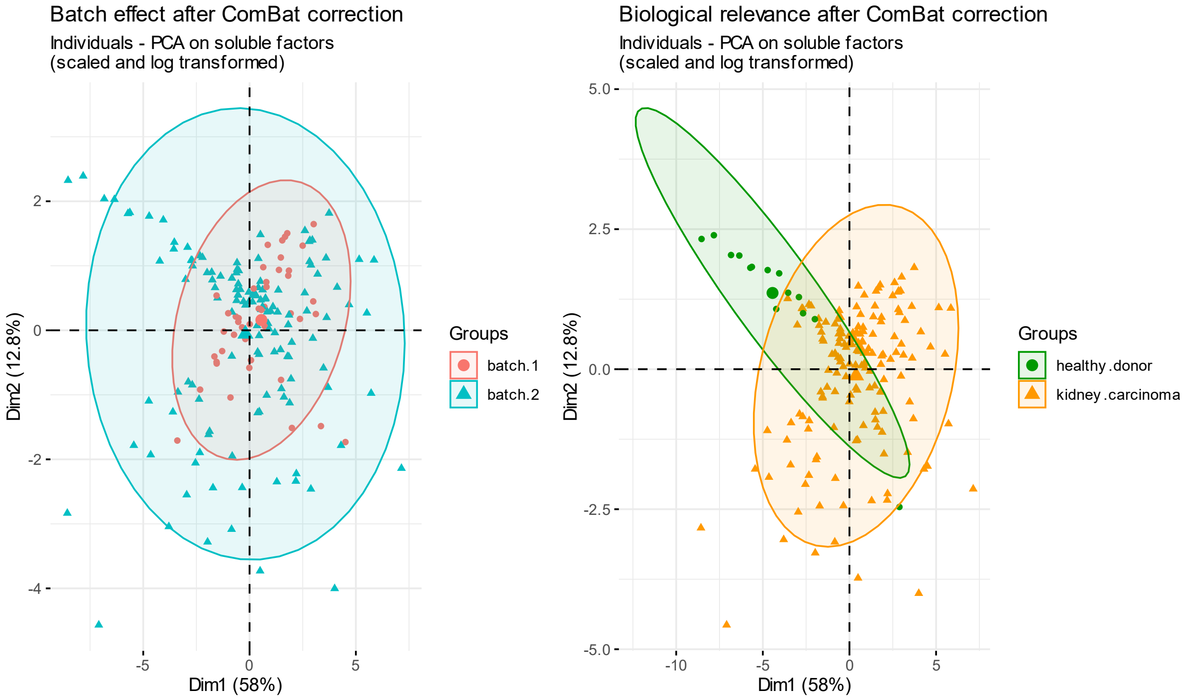

Supp.Fig.1: PCA of batch-corrected soluble factors values for participants of the BIONIKK study. *In order to minimize the influence of a potential batch effect of the Luminex quantification method, sTIM3 values were batch-corrected through the ComBat() function from the sva R package (v. 3.50.0) prior to classification of individuals for comparisons between the nivolumab and N+I arms of the BIONIKK cohort. This method enabled to successfully limit technical variability between batches, while preserving biological significance. As a control, healthy donors were clearly distinguished from ccRCC patients after dimensional reduction of the data of other soluble proteins quantified along sTIM-3 in these individuals*

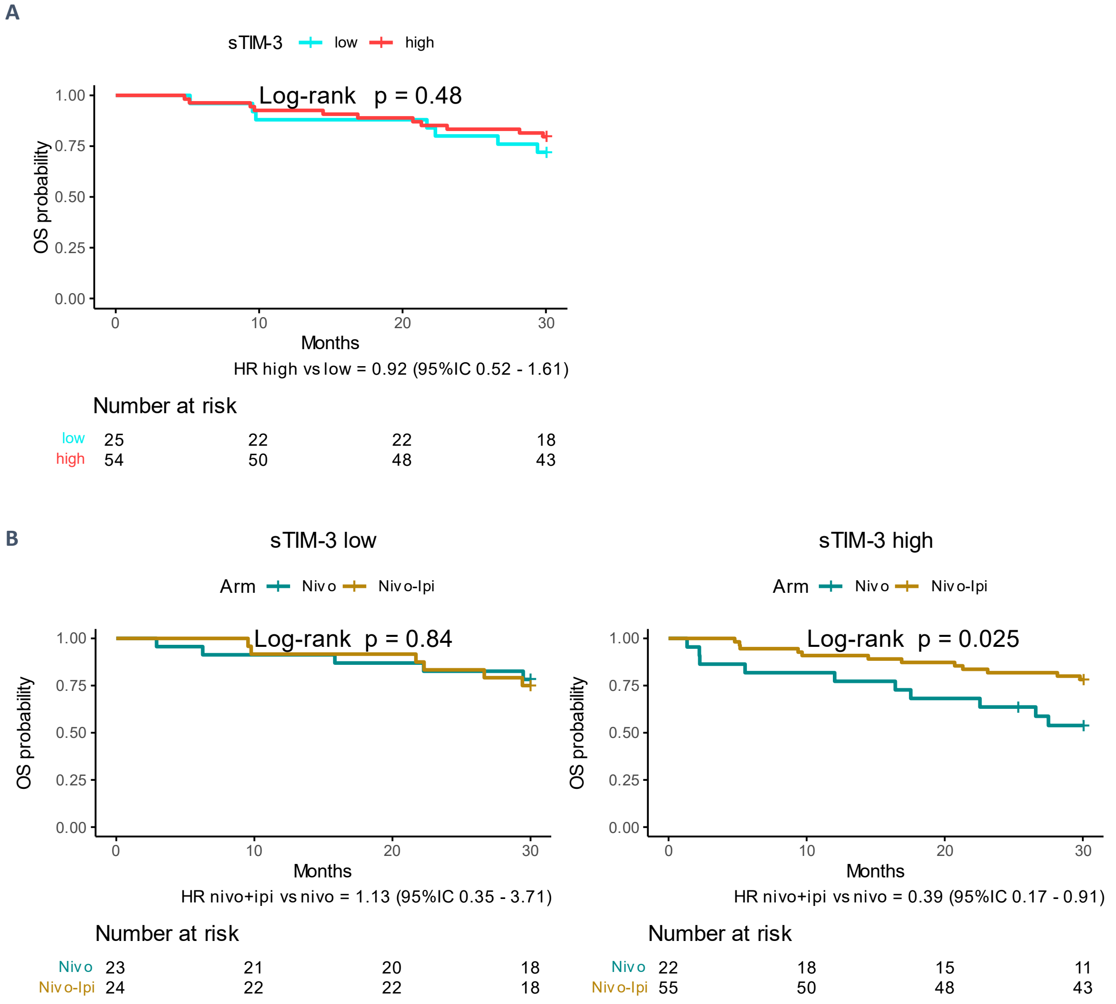

Sup.Fig.2: Association of plasma sTIM-3 and OS in patient from the BIONIKK trial treated with nivolumab + ipilimumab combination. A. OS in sTIM-3 high vs sTIM-3 low mccRCC patients treated with nivolumab + ipilimumab and **B.** Comparison of OS between nivolumab + ipilimumab and nivolumab monotherapy within each sTIM-3 strata.

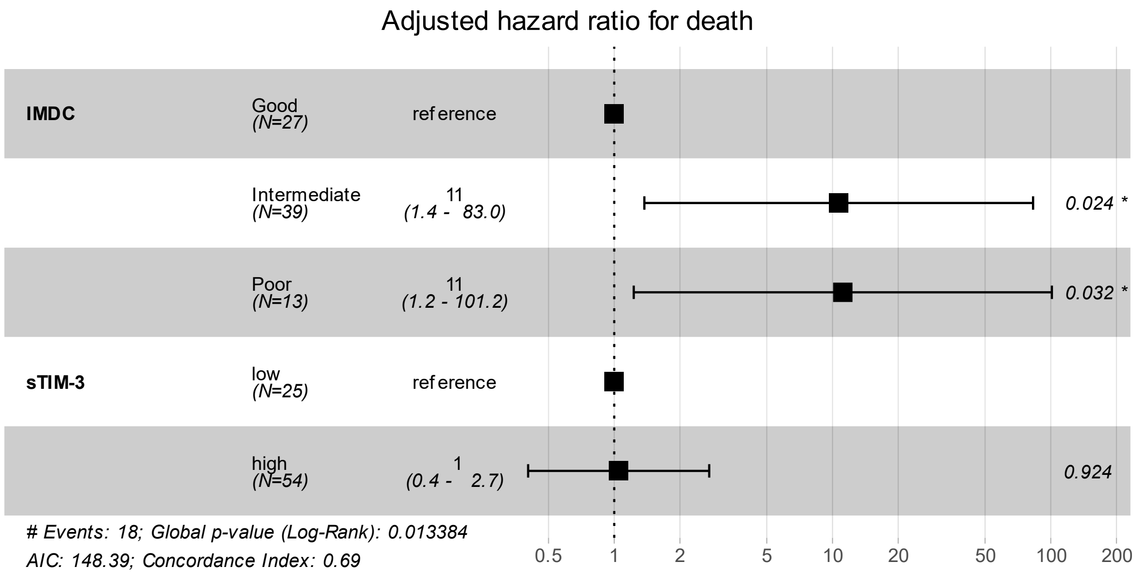

Supp.Fig.3: **Multivariable Cox regression model for OS, adjusting for IMDC in BIONIKK nivolumab + ipilimumab participants.**

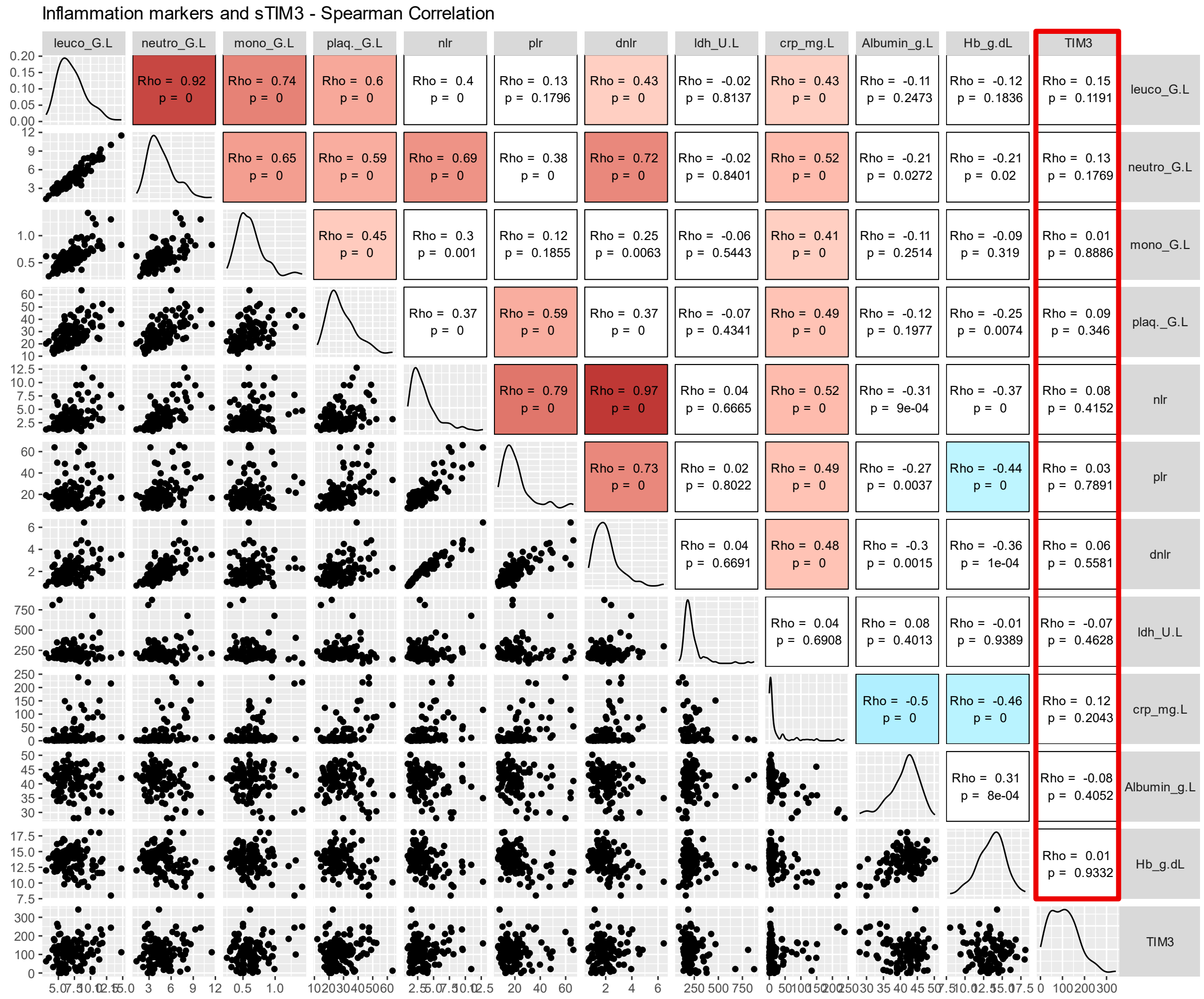

Supp.Fig.4: **Spearman correlation matrix between classical inflammatory markers and sTIM-3**

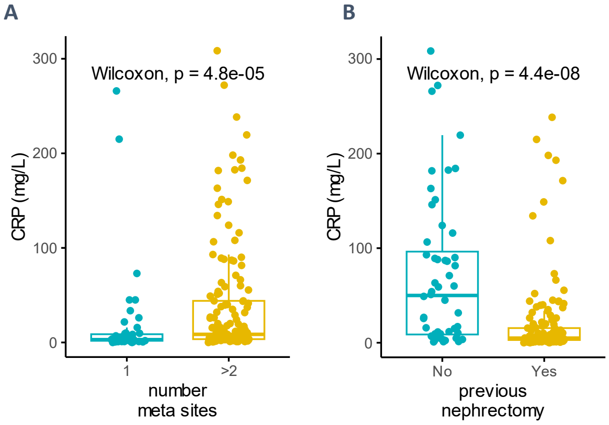

Supp.Fig.5: **Comparisons of serum CRP in BIONIKK participant. A.** 1 versus ≥2 metastatic sites. **B.** Primary tumor resected (“previous nephrectomy yes”) versus not resected.

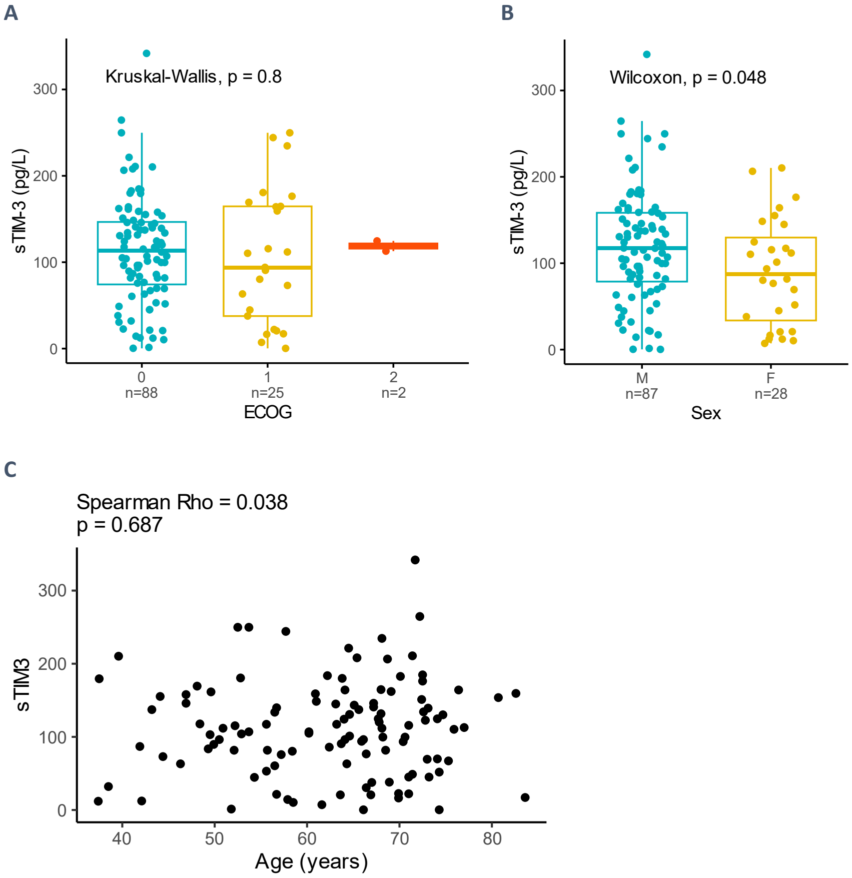

Supp.Fig.6: Association of sTIM-3 with clinical variables in BIONIKK participants: A. sTIM-3 versus ECOG performance status. **B.** sTIM-3 versus sex. **C.** sTIM-3 versus age.

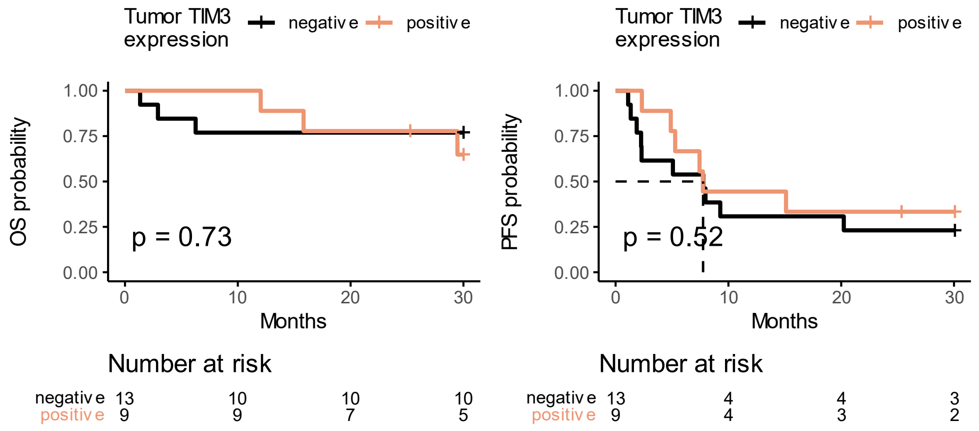

Supp.Fig.7: OS (left) and PFS (right) in the BIONIKK IHC subgroup of participants treated with Nivolumab monotherapy stratified according to tumor TIM-3 staining.

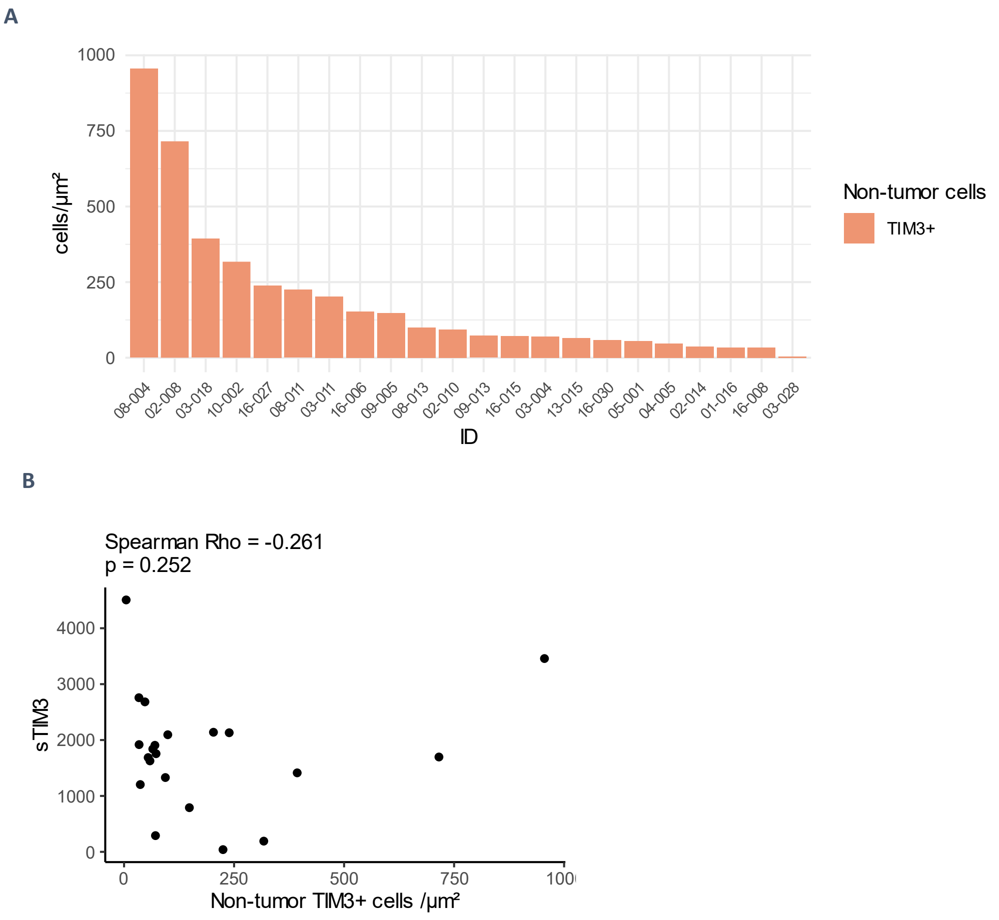

Supp.Fig.8: Membrane TIM-3 on non-tumor cells in IHC. A. Density of non-tumor TIM-3 positive cells assessed by IHC in tumors of the BIONIKK patients IHC subset. **B.** Correlation plot between non-tumor TIM-3 positive cells density and sTIM-3 plasmatic levels

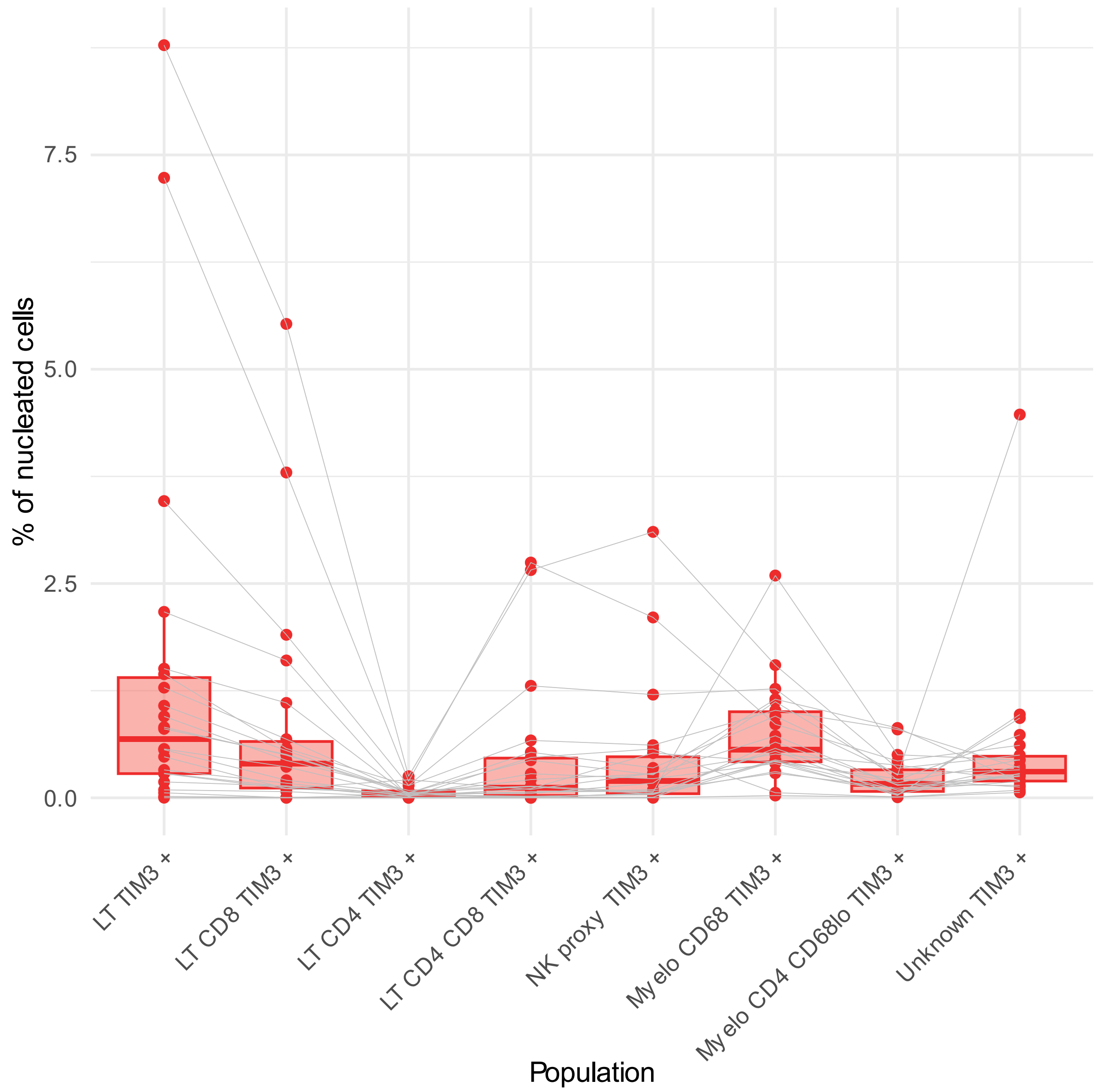

Supp.Fig.9: **Membrane TIM-3 on non-tumor cells in IHC, detailed subtypes.** TIM-3positive cells are quantified as % of all nucleated cells detected on the area of analysis. LT = T cells; LT CD4 CD8 = double-positive CD4+CD8+T cells ; NK proxy = CD3-CD8+cells ; Myelo CD4 CD68lo = CD3-CD4+CD68low/-cells; unknown = TIM-3-positive cells that were not positive for any other marker from the IHC panel.

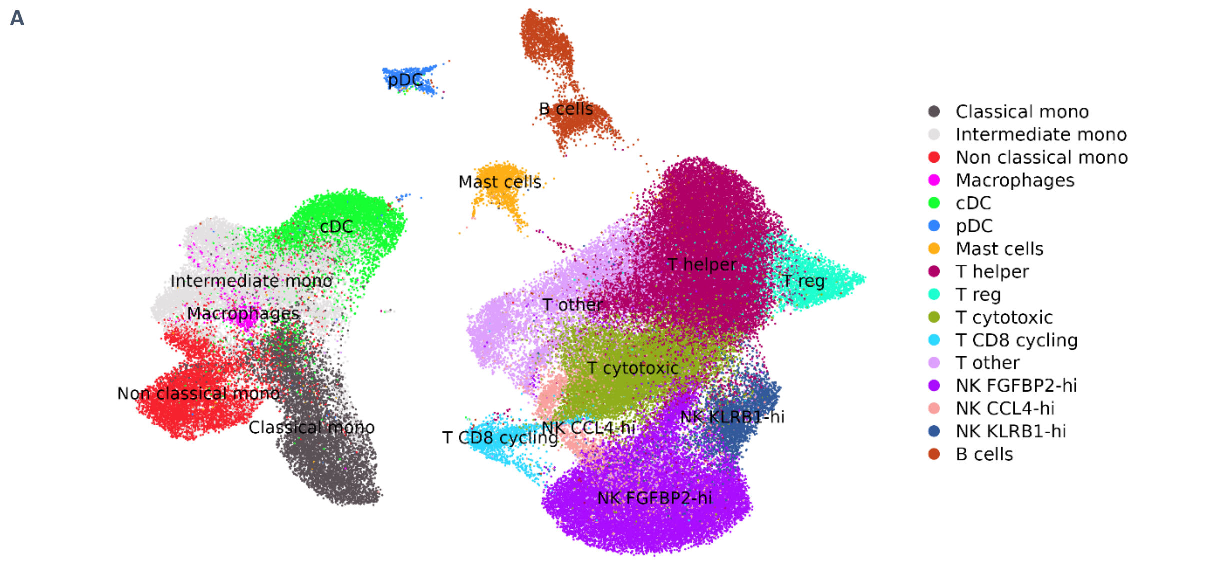

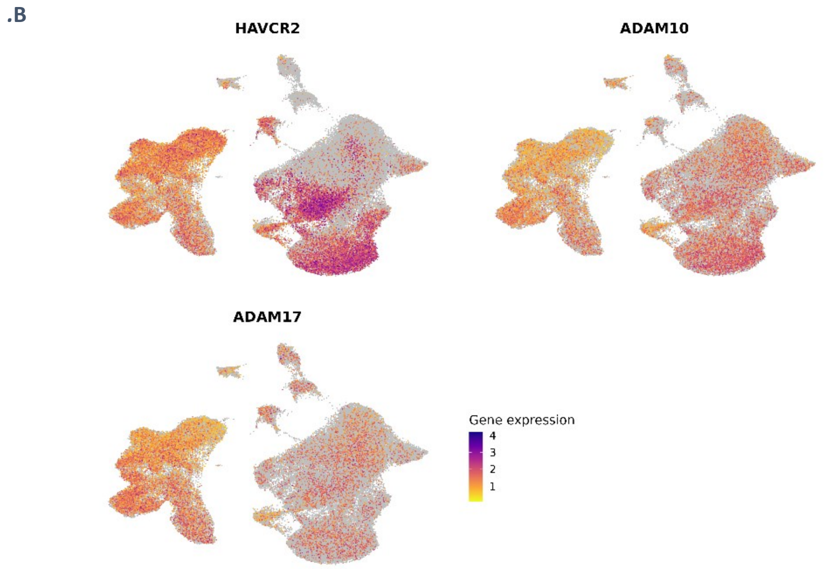

Supp.Fig.10: **UMAPs of CD45 positive cells from the scRNAseq dataset of Obradovic et al. A.** Detailed clusters. **B.** Gene expression levels for HAVCR2, ADAM10 and ADAM17. **C.** Detail of HAVCR2 and ADAM10/17 co-expression phenotypes.

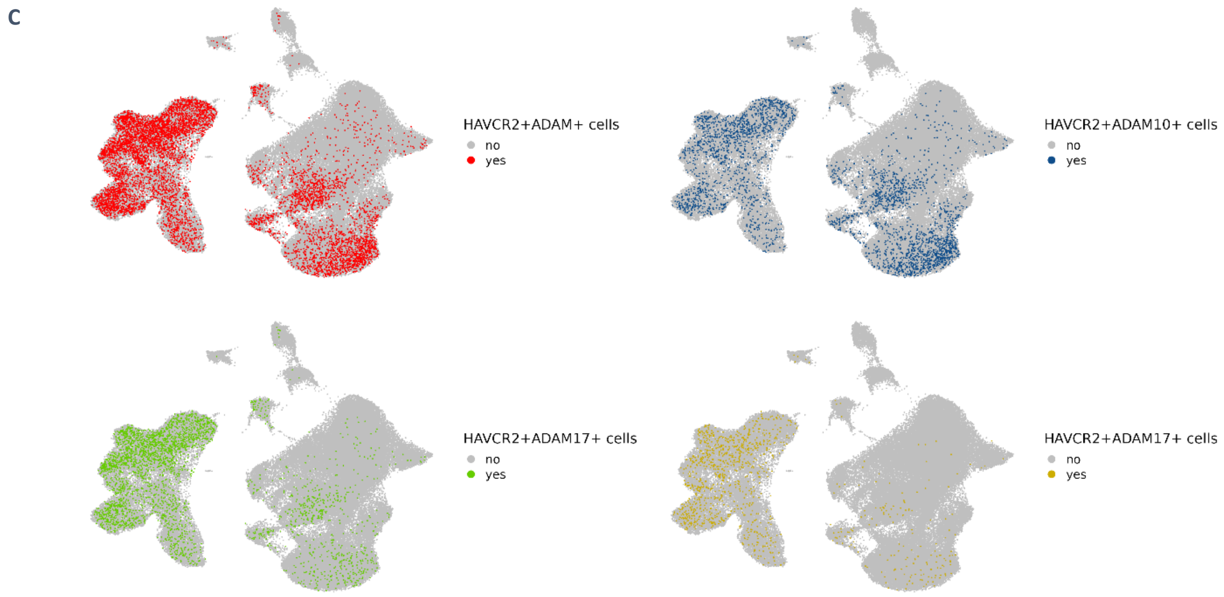

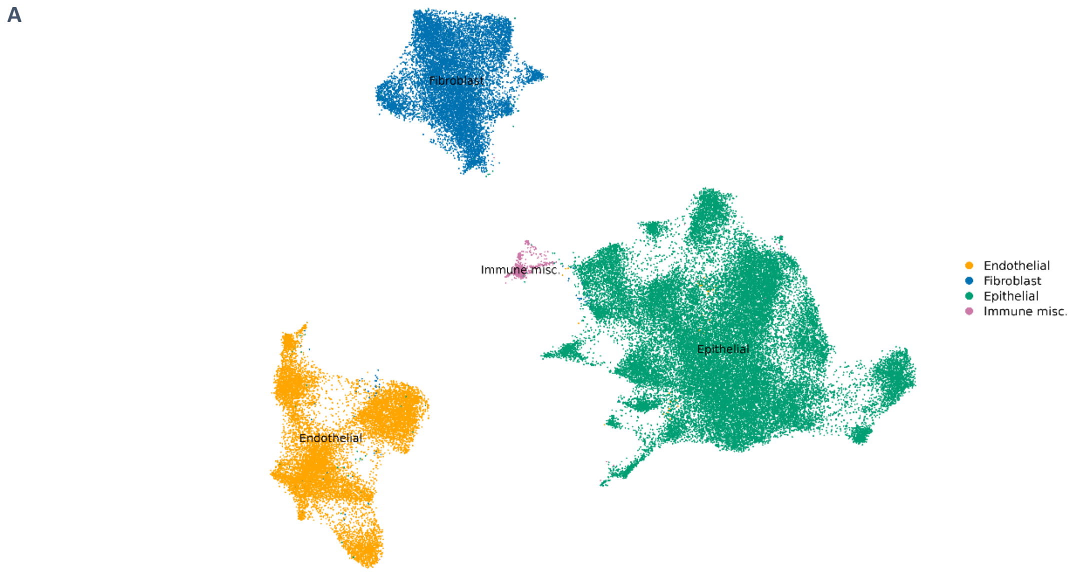

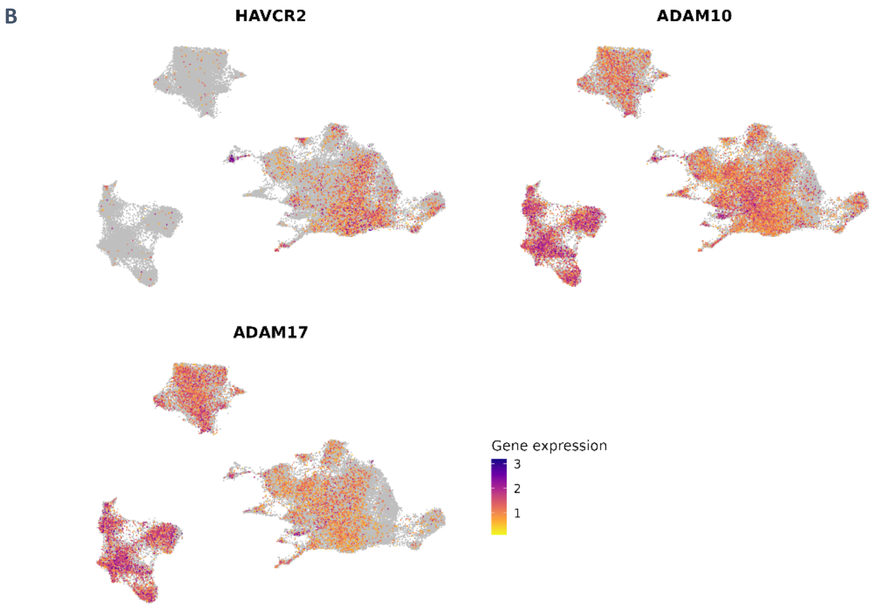

Supp.Fig.11: **UMAPs of CD45 positive cells from the scRNAseq dataset of Obradovic et al. A.** Detailed clusters. **B.** Gene expression levels for HAVCR2, ADAM10 and ADAM17. **C.** Detail of HAVCR2 and ADAM 10/17 co-expression phenotypes.

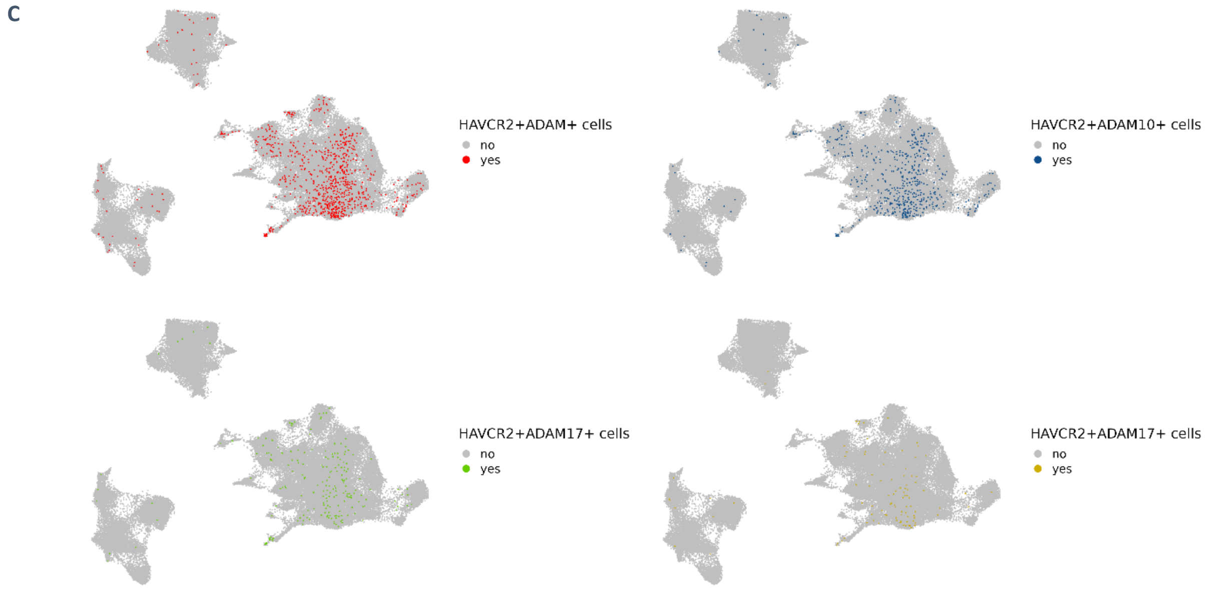

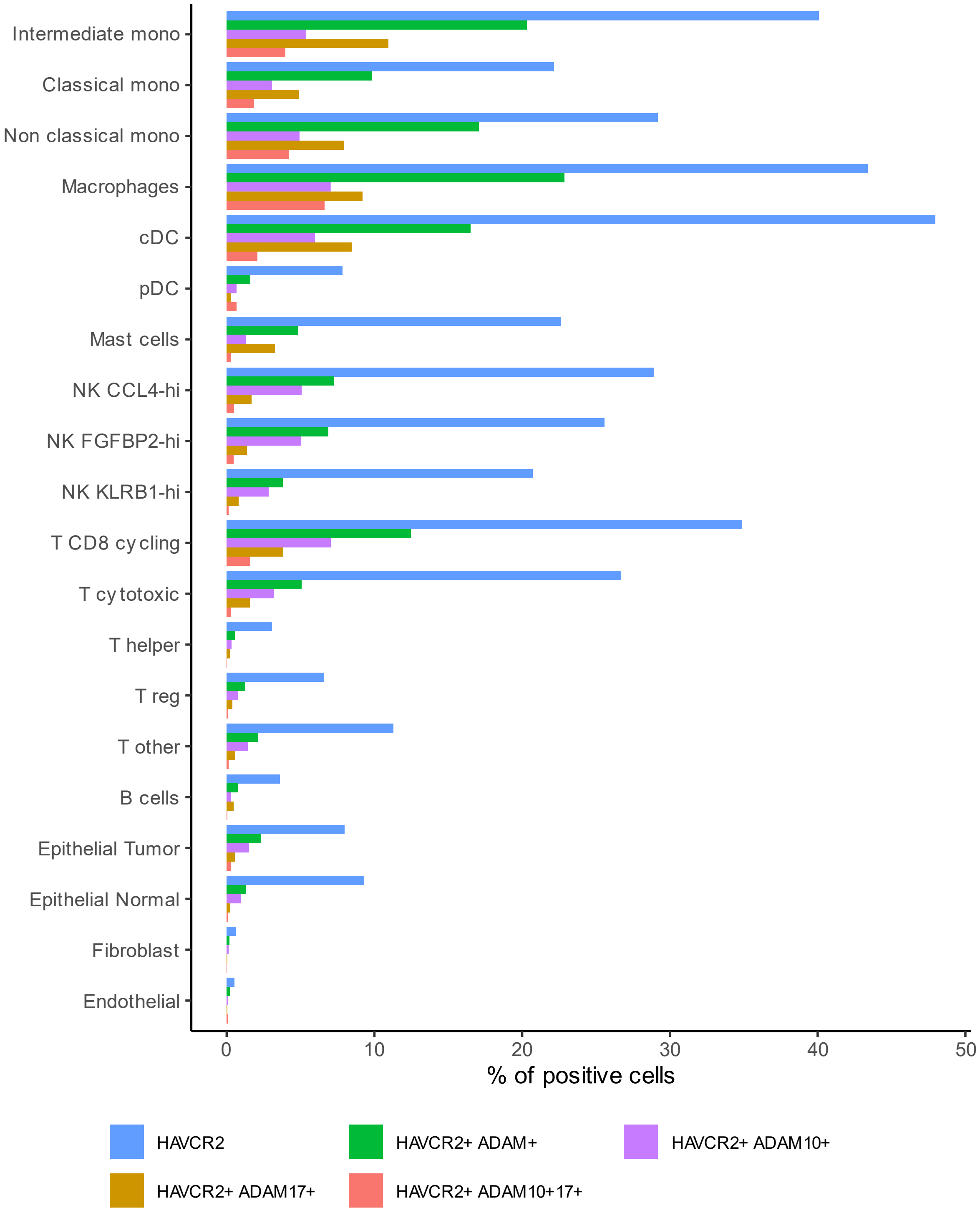

Supp.Fig.12: **Percentage of cells expressing HAVCR2 or co-expressing HAVCR2, ADAM10, ADAM17, within each cluster in the scRNAseq dataset of Obradovic et al.**

A

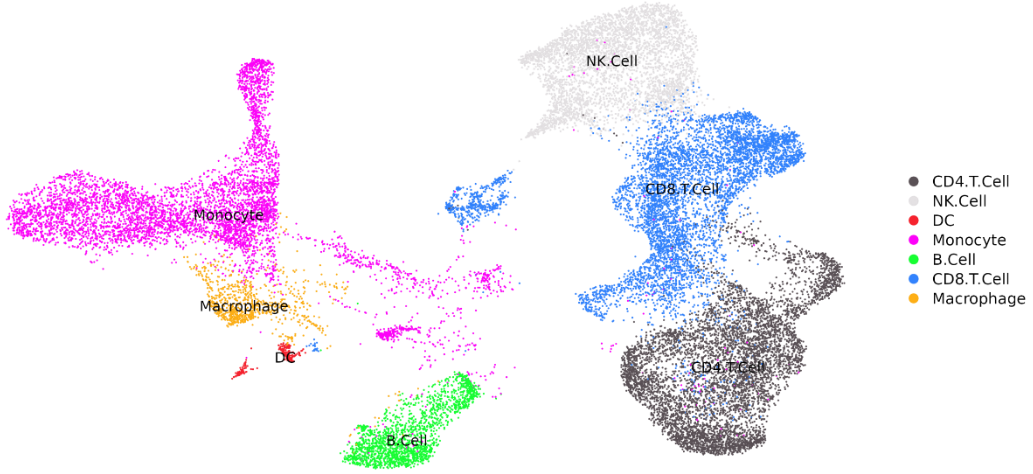

B

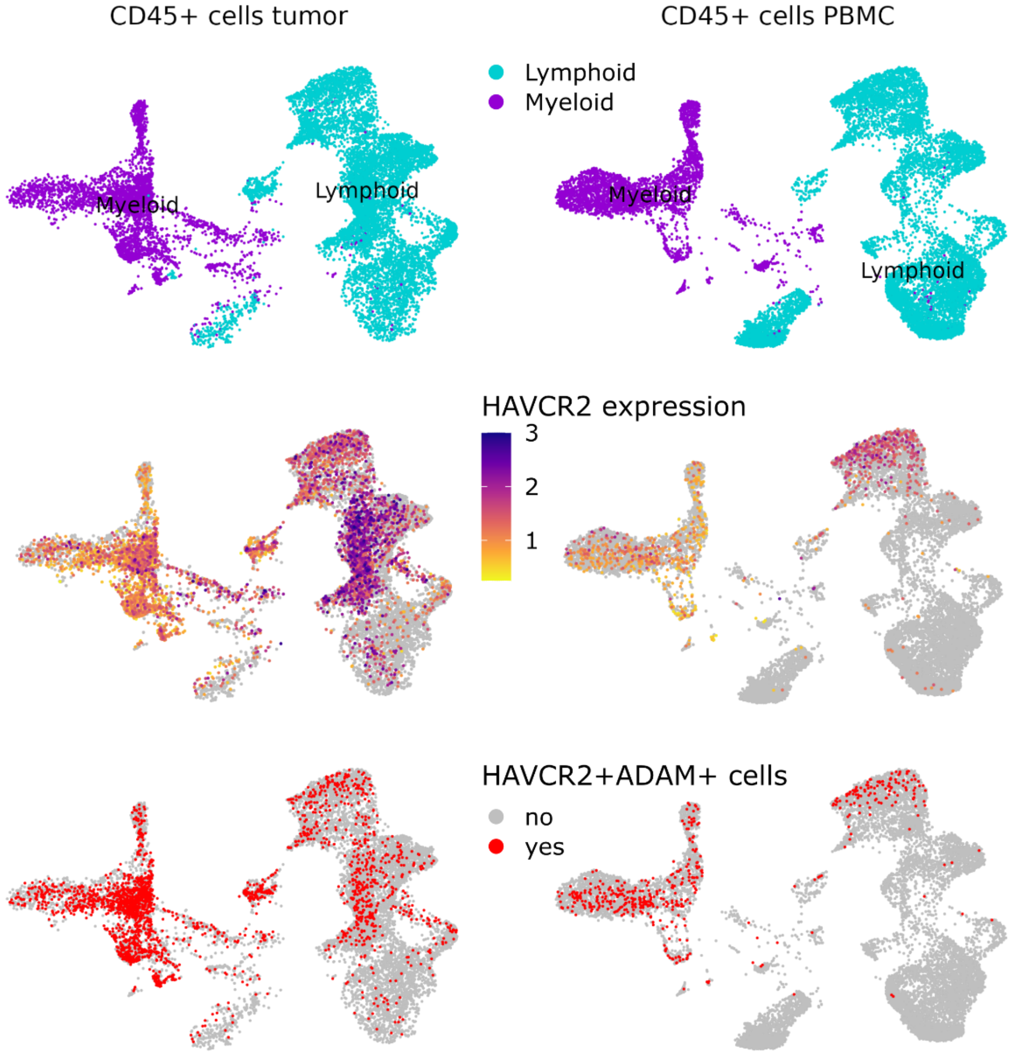

Supp.Fig.13: **UMAPs of CD45 positive cells from 3 ccRCC patients in the scRNAseq dataset of Borcherding et al.** **A.** Detailed clusters **B.** Comparison of immune cells from matched tumor and PBMC samples. upper section: UMAP of cell lineages. middle section: HAVCR2 expression. lower section: HAVCR2+ADAM+ double-positive cells repartition

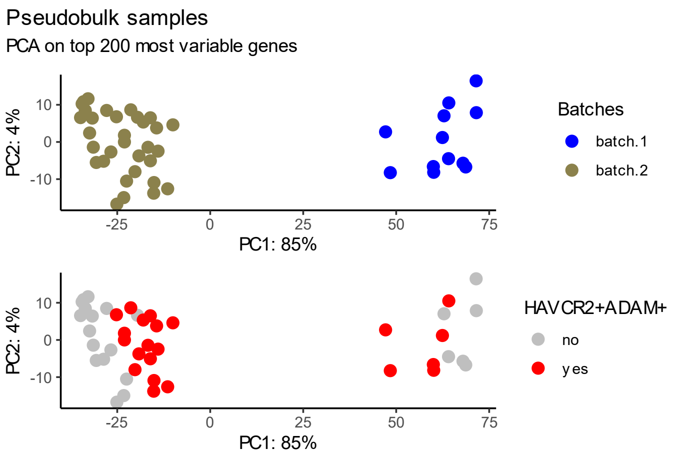

Supp.Fig.14: **Pseudobulk samples of HAVCR2+ADAM+ double-positive and non-double-positive myeloid cells from Obradovic et al. scRNAseq dataset. Projection of samples on the first two axes of PCA on the top 200 most variable genes.** Transcripts counts cells were aggregated in pseudobulk samples to compare HAVCR2+ADAM+ myeloid cells to other myeloid cells. Top panel: visualization of the batch effect. Bottom panel: segregation of double-positive and non-double-positive pseudobulk samples within each batch.

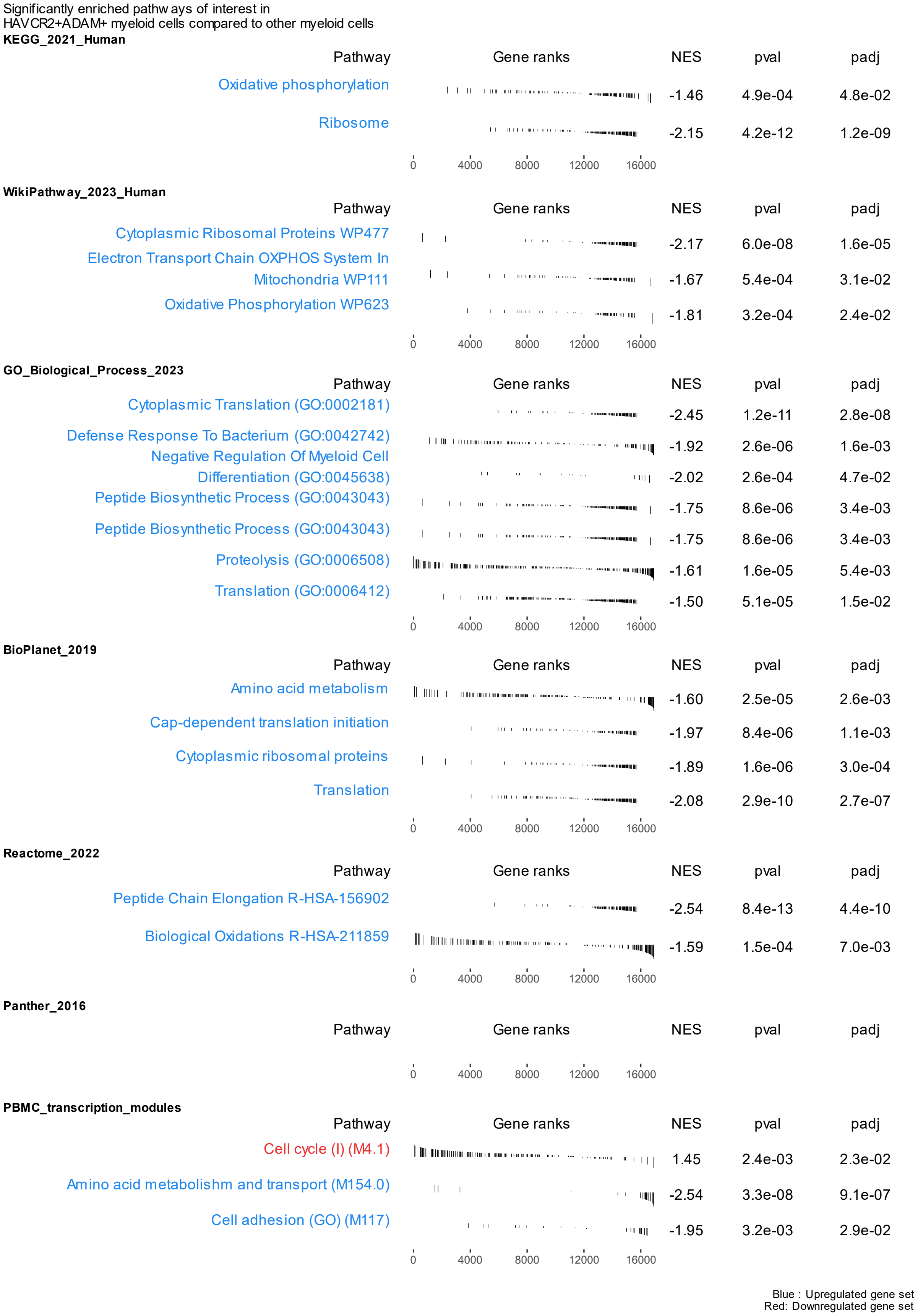

Supp.Fig.15: Pathways of immunological interest with significant enrichment after GSEA on differentially expressed genes between HAVCR2+ADAM+ myeloid cells and other myeloid cells from Obradovic et al. NES: normalized enrichments score. Padj: adjusted p-value.

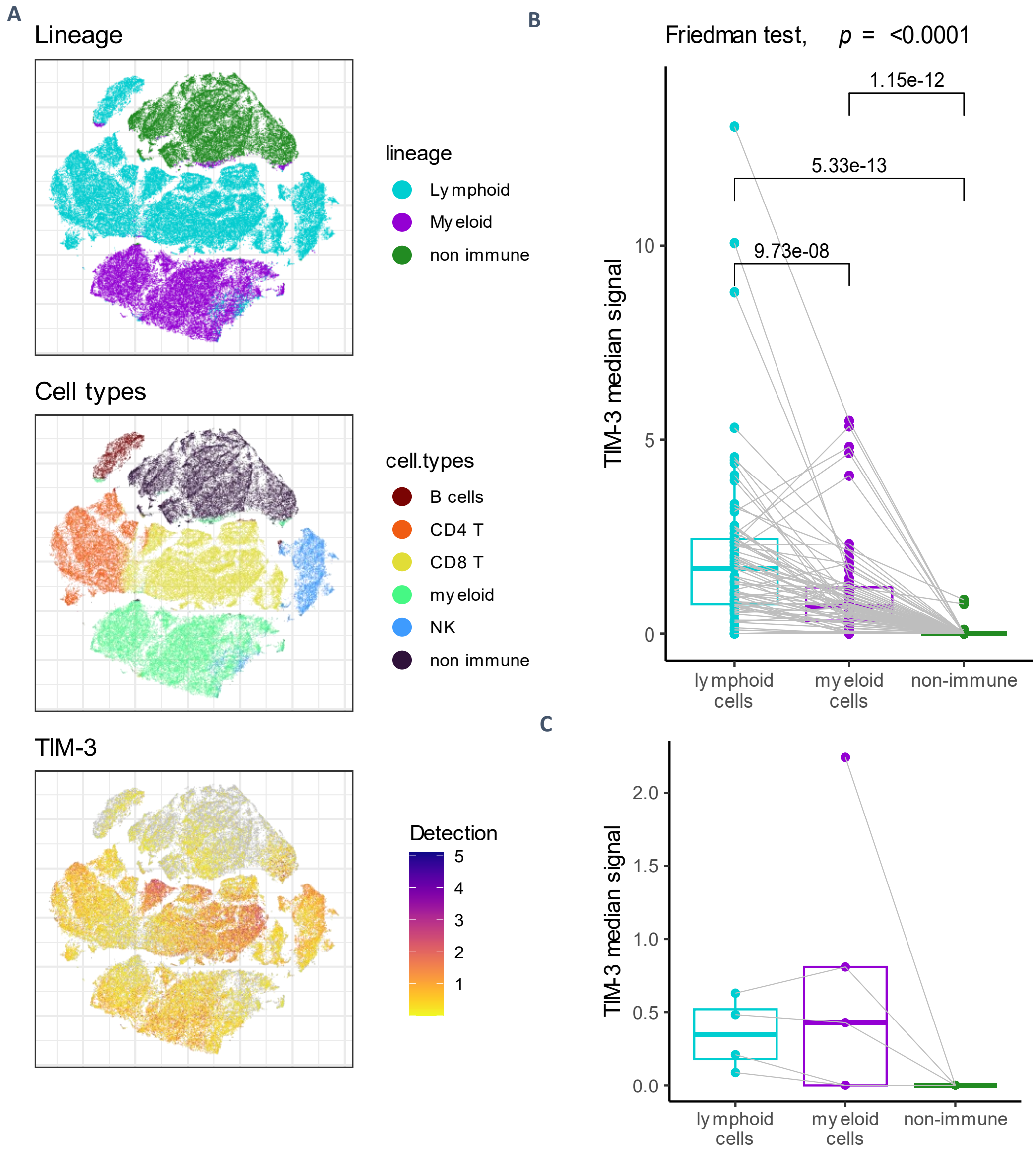

Supp.Fig.16*: Mass cytometry quantification of TIM-3-expression cells in the dataset of Chevrier et al.* of 72 ccRCC tumors and 5 healthy kidney samples. *Publicly available mass cytometry "T cell panel" data from Chevrier et al. was accessed at https://premium.cytobank.org/cytobank/projects/875* (25). *.fcs files correspond to debarcoded samples of viable cells, filtered out for gadolinium contamination and cells doublets. The data is bead-based normalized. We applied the standard arcsinh transformation with a cofactor of 5 on the value of all channels for clustering and visualization purposes. A compensation matrix was calculated with the CATALYST R package (v. 1.18.1) according to the procedure and single-stained beads reference assays reported by Chevrier et al.* (44)*. Given the absence of significant changes after applying compensation (**Supp.Fig.17**), the data was left uncompensated. Annotation of cells by broad lineages and immune cell types was performed by unsupervised k-means clustering and examination of the expression profile of consensus markers. Contrary to the procedure used by Chevrier et al., markers such as TIM-3 and other immune checkpoint were not included in the matrix used to cluster cells, to avoid the artificial construction of TIM-3-high clusters. The data was scaled prior to k-means clustering and a ponderation was used with higher weights attributed to major markers such as CD45 and CD3. The expression of TIM-3 and other markers on single cells was visualized on dimensionality reduction 2-D projections generated with the t-SNE algorithm, after subsampling the*

whole dataset to a maximum of 1500 cells per sample for convenience. For statistical comparisons, the median signal intensity of TIM-3 was calculated on untransformed values.

A. tSNE representation of cell clusters and TIM-3 expression (asinh transformed values) after subsampling of the dataset. **B.** Median signal intensity (untransformed) for TIM-3 in lymphoid, myeloid and non-immune cells of the ccRCC samples. P-values are given on brackets for paired Wilcoxon tests between each lineage. **C.** Median signal intensity (untransformed) for TIM-3 in lymphoid, myeloid and non-immune cells of the healthy donors' samples.

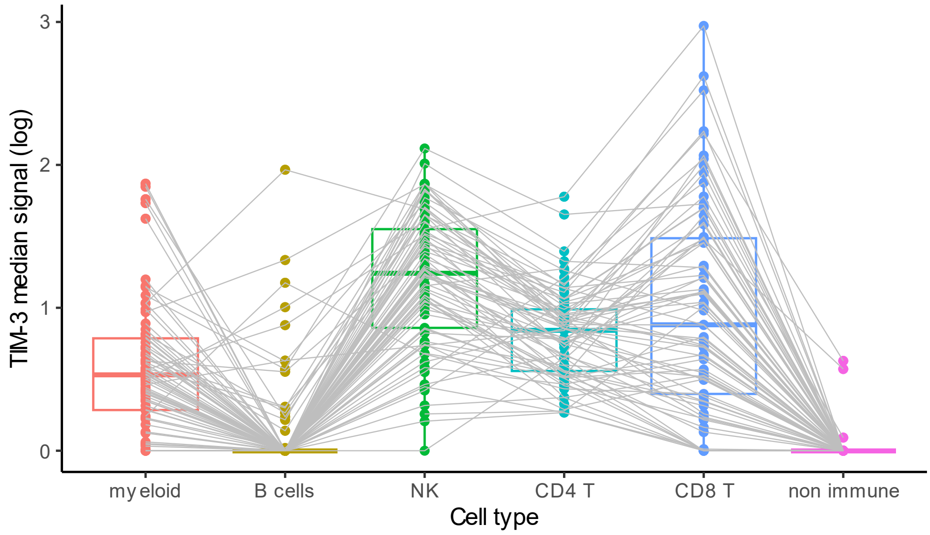

Supp.Fig.17: **Median signal intensity for TIM-3 in immune and non-immune cells of the ccRCC samples from Chevrier et al. dataset.** Same as **Supp.Fig.16.B** with finer clustering. In this figure, the median signal intensity for TIM-3 is added to 1 and then converted to log values for plotting convenience.

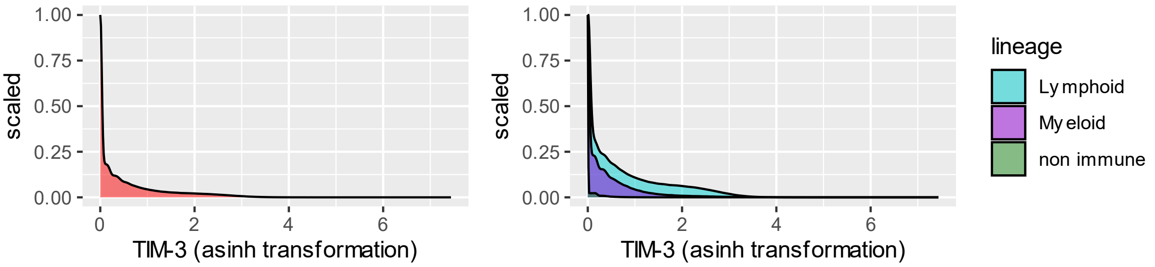

Supp.Fig.18: **TIM-3 detection density plots on the mass cytometry dataset of Chevrier et al..** Values are asinh-transformed. Left: all cells. Right: By lineage

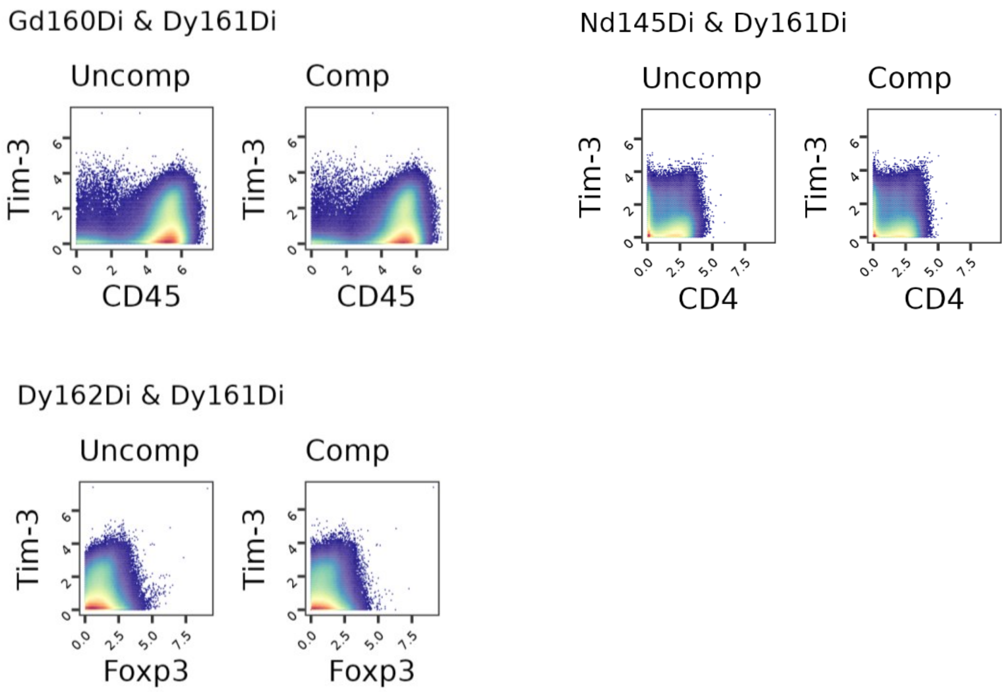

Supp.Fig.19: **Contour plots visualizing the effect of spillover compensation on TIM-3 signal measure and potentially interacting mass channels.** Values are asinh-transformed. “Uncomp” = uncompensated values. “Comp” = after compensation.

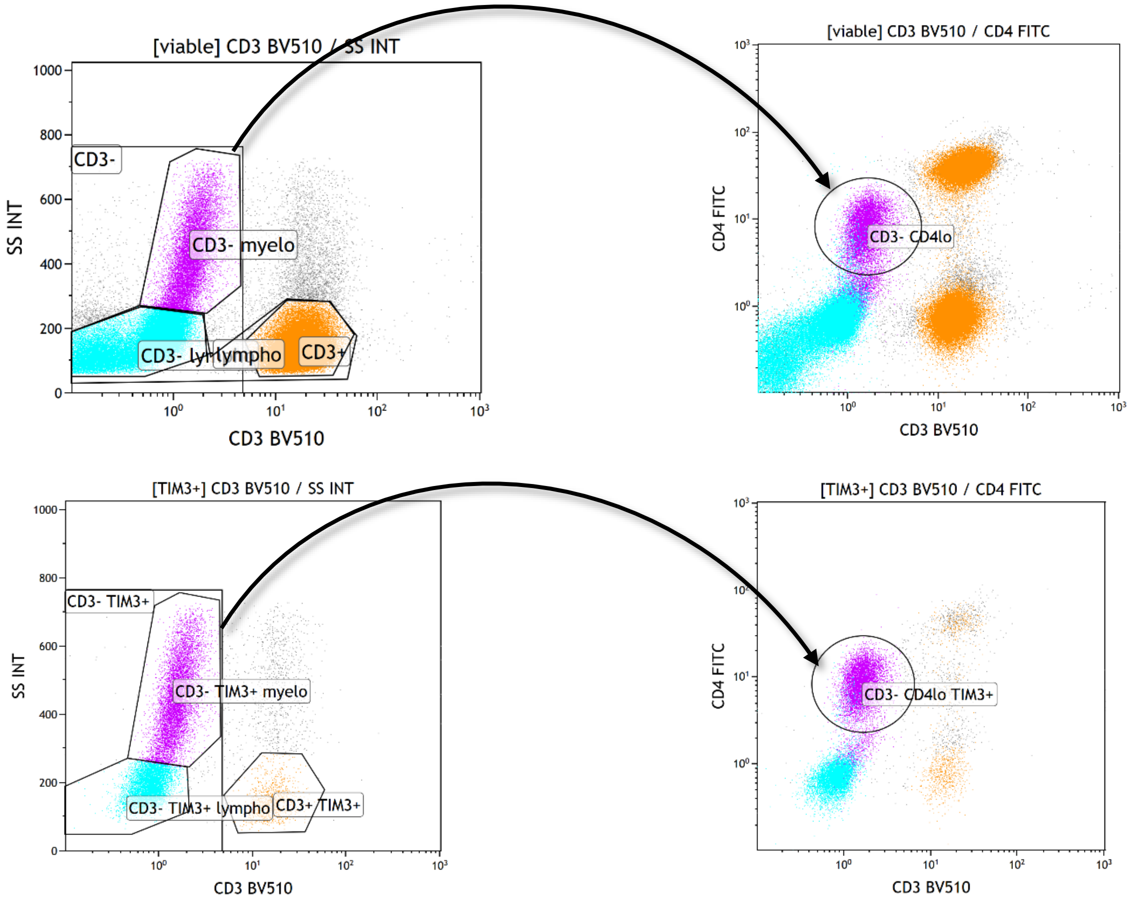

Supp.Fig.20: **Representative flow cytometry plot showing intermediate levels of CD4 expression** by the "CD3- myeloid" (top left and right, gated on viable cells) and "TIM3+CD3- myeloid" (bottom left and right, gated on TIM3+ cells) populations.

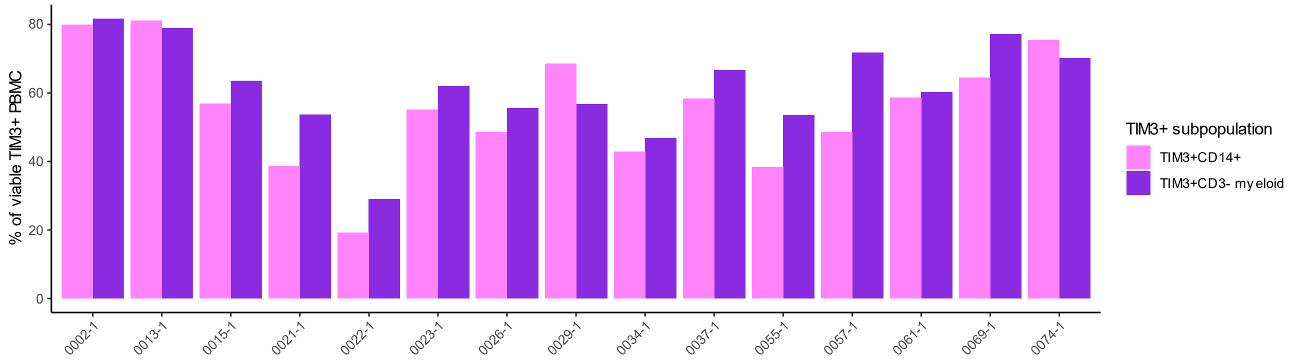

Supp.Fig.21: **Head-to-head comparison of the CD14+ and CD3- myeloid cells proportions within TIM3+ PBMC**, showing the correspondence of the two populations in a subset of patients from the Colcheckpoint cohort (n = 15), for which an additional cytometry panel with CD14 was performed on the same PBMC sample.

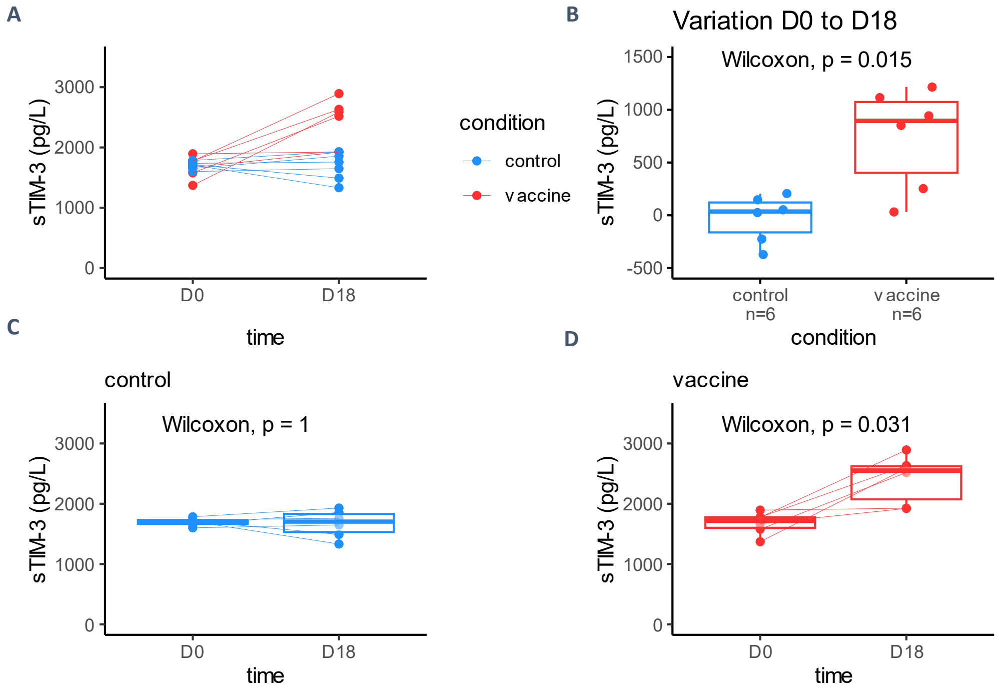

Supp.Fig.22: **sTIM-3 plasma levels in TC-1 syngeneic mice immunized with an antitumor vaccine**. **A.** TC-1 syngeneic mice were inoculated (intra-jugal, 50,000 cells/50 µL) with TC-1 tumor cells (murine pulmonary epithelial line immortalized by the E6 and E7 proteins from human papillomavirus 16 – HPV-16) at day 0 (D0) and then vaccinated (vaccine group, injection at D7 and D14) or not (control group), plasma sTIM-3 was measured at D0 and D18, n = 6 individuals for both groups. **A.** All measures. **B.** Comparison of sTIM-3 variation (D18 values minus D0 value for each individual) between control and vaccine group (Wilcoxon rank sum test). **C & D.** Measure of sTIM-3 levels over time within control and vaccine groups respectively (Wilcoxon matched pairs test).

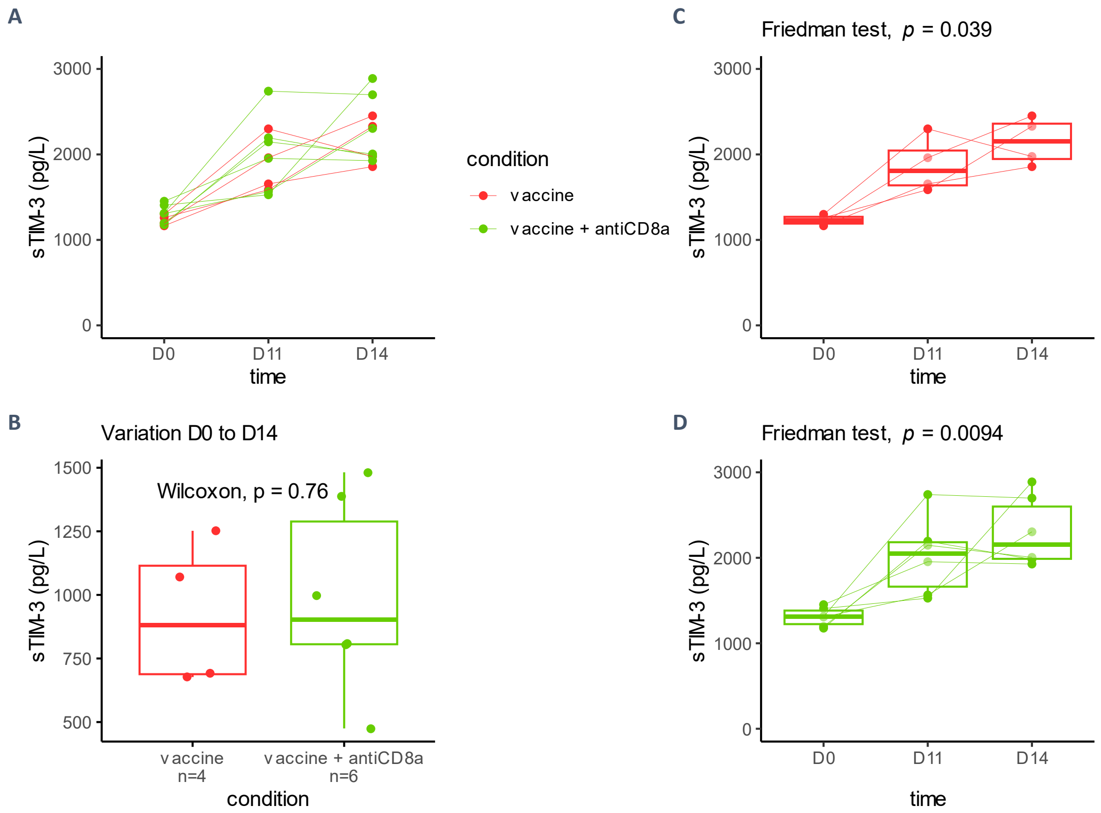

Supp.Fig.23: sTIM-3 plasmatic levels in syngeneic mice inoculated with TC-1 cells, immunized with an antitumor vaccine and ± depleted for CD8+ T cells. red: vaccinated (at D7, n= 4); green: vaccinated + anti-CD8α (vaccine at D7, anti-CD8α at D5 and D12; n= 5). Serum sTIM-3 measures are given in pg/L **A.** All measures. **B.** Comparison of serum sTIM-3 variation between day 0 (D0, tumor graft) and day 14 (D14) in vaccinated versus vaccinated + CD8 T cells depleted mice. **C & D.** serum sTIM-3 evolution over time in each group.

Supplementary tables

A.

Variable	Categories	sTIM-3-low	sTIM-3-high	p
**n**		14	13	
**Age (mean (SD))**		60.59 (13.96)	68.99 (10.26)	0.089
**Sex (%)**	F	0 (0.0)	5 (38.5)	0.016
	M	14 (100.0)	8 (61.5)	
**ECOG PS (%)**	0	10 (71.4)	10 (76.9)	0.648
	1	4 (28.6)	2 (15.4)	
	2	0 (0.0)	1 (7.7)	
**IMDC (%)**	Good	6 (42.9)	4 (30.8)	0.695
	Intermediate	8 (57.1)	8 (61.5)	
	Poor	0 (0.0)	1 (7.7)	
**Previous nephrectomy (%)**	no	4 (28.6)	2 (15.4)	0.648
	yes	10 (71.4)	11 (84.6)	

B.

Variable	Categories	sTIM-3-low	sTIM-3-high	p
**n**		22	23	
**Age (mean (SD))**		59.95 (8.14)	61.42 (10.19)	0.595
**Sex (%)**	F	9 (40.9)	5 (21.7)	0.208
	M	13 (59.1)	18 (78.3)	
**ECOG PS (%)**	0	16 (72.7)	15 (65.2)	0.816
	1	4 (18.2)	4 (17.4)	
	2	2 (9.1)	4 (17.4)	
**IMDC (%)**	Good	7 (31.8)	2 (8.7)	0.052
	Intermediate	11 (50.0)	10 (43.5)	
	Poor	4 (18.2)	11 (47.8)	
**Previous nephrectomy (%)**	No	7 (31.8)	6 (26.1)	0.749
	Yes	15 (68.2)	17 (73.9)	

C.

Variable	Categories	sTIM-3-low	sTIM-3-high	p
**n**		25	54	
**Age (mean (SD))**		61.83 (9.63)	63.15 (10.54)	0.597
**Sex (%)**	F	6 (24.0)	10 (18.5)	0.563
	M	19 (76.0)	44 (81.5)	
**ECOG PS (%)**	0	20 (80.0)	43 (79.6)	0.889
	1	5 (20.0)	9 (16.7)	

	2	0 (0.0)	2 (3.7)	
**IMDC (%)**	Good	4 (16.0)	23 (42.6)	**0.045**
	Intermediate	17 (68.0)	22 (40.7)	
	Poor	4 (16.0)	9 (16.7)	
**Previous nephrectomy (%)**	No	6 (24.0)	11 (20.4)	0.772
	Yes	19 (76.0)	43 (79.6)	

Supp.Table 1: **Characteristics of participants analyzed for OS, stratified on sTIM-3 categorization in A.** Colcheckpoint. **B.** BIONIKK nivolumab-treated. **C.** BIONIKK nivolumab-ipilimumab treated.

A.

**Target**	**Manufacturer**	**Reference**	**Native concentration**	**Dilution**
**TIM-3**	CST®	45208	16 µg/mL	1:300
**PAX8**	Abcam®	ab191870	853 µg/mL	1:400
**pan-Cytokeratin**	CST®	4545	190 µg/mL	1:250
**CD3**	Bethyl®	A700-016	50 µg/mL	1:300
**CD8**	Bethyl®	A700-044	250 µg/mL	1:250
**CD4**	Bethyl®	A700-015	1000 µg/mL	1:250
**CD68**	Dako®	M0814	185 µg/mL	1:800

B.

**Target**	**Manufacturer**	**Reference**	**Fluorophore**
**CD3**	BioLegend®	300448	BV510
**CD4**	BioLegend®	300506	FITC
**TIM-3**	BioLegend®	345006	PE
**CD8**	BioLegend®	301006	FITC
**CD14**	BioLegend®	367124	BV510
**CD19**	BioLegend®	302206	FITC

Supp.Table 2: Antibodies used for multiplex IHC and flow cytometry experiments. A. IHC antibodies. **B.** Flow cytometry antibodies. Staining was performed with 3 separate panels: 1st panel: anti-CD3, anti-CD4, anti-TIM-3; 2nd panel: anti-CD3, anti-CD8, anti-TIM-3; 3rd panel: anti- CD14, anti-CD19, anti-TIM-3.
